# Supplementary material for: Septins Recognize and Entrap Dividing Bacterial Cells for Delivery to Lysosomes
Source: Cell Host Microbe. 2018 Dec 12;24(6):866–874.e4. doi: 10.1016/j.chom.2018.11.005 (PMC6299245; doi:10.1016/j.chom.2018.11.005)
Supplement: Document S1. Figures S1–S4 and Table S1 [file mmc1.pdf]

Cell Host & Microbe, Volume *24*

Supplemental Information

Septins Recognize and Entrap Dividing Bacterial Cells for Delivery to Lysosomes

Sina Krokowski, Damián Lobato-Márquez, Arnaud Chastanet, Pedro Matos Pereira, Dimitrios Angelis, Dieter Galea, Gerald Larrouy-Maumus, Ricardo Henriques, Elias T. Spiliotis, Rut Carballido-López, and Serge Mostowy

Cell Host & Microbe

Supplemental Information

Septins Recognise Dividing Bacterial Cells for Delivery to the Lysosome

Sina Krokowski, Damián Lobato-Márquez, Arnaud Chastanet, Pedro Matos Pereira, Dimitrios Angelis, Dieter Galea, Gerald Larrouy-Maumus, Ricardo Henriques, Elias T. Spiliotis, Rut Carballido-López, Serge Mostowy

Figure S1. Septins Recognise Micron-Scale Bacterial Curvature, Related to Figure 1.

(A) Illustrating the engineering of a plasmid-encoded (pBAD18), arabinose-controlled FtsZ-monomeric superfolder green fluorescent protein (GFP) fusion in *S. flexneri*.

(B) Localisation of FtsZ-GFP in *S. flexneri* in broth with respect to membrane (FM4-64X) and DNA (DAPI). Scale bar = 1 µm.

(C) Length and width of WT and FtsZ-GFP overproducing *Shigella*. Graph shows median and whiskers (min to max) from n = 600 bacterial cells from 3 independent experiments. Student's t-test, ns $p > 0.05$.

(D) Growth of *Shigella* WT, Vector (empty pBAD18) and FtsZ-GFP was determined by measuring the optical density (OD_{600}) every 30 min for 15 h using a microplate reader. Graph shows mean OD_{600} ± SEM from 7 independent experiments. Student's t-test on last time point, ns $p > 0.05$.

(E) HeLa cells were infected with *Shigella* WT, Vector (empty pBAD18) or FtsZ-GFP and the intracellular bacterial load (CFU / mL) was determined 1 h and 4 h 40 min post infection. Invasion of host cells and intracellular survival is not affected by pBAD18 or FtsZ-GFP. Graph shows mean CFU / mL ± SEM from 3 independent experiments. Student's t-test, ns $p > 0.05$.

(F) *S. flexneri* FtsZ-GFP infected U-2 OS cells at 3 h 40 min post infection. Single-particle analysis of 28 SIM images of *Shigella*-septin cages with SEPT7 localising to FtsZ. Scale bar = 1 µm. Inset images highlight a conserved septin ring at the bacterial division site. Fluorescent intensity profile (FIP) was taken of the dotted line along the midline of the cell in the inset image and normalised from 0 to 1.

(G) Representative three-dimensional SIM images (top) and diagram (bottom) of a SEPT7 ring surrounding the bacterial Z-ring.

(H) Time-lapse SIM of *S. sonnei*-mCherry infected SEPT6-GFP HeLa cells at 1 h 40 min post infection imaged every 2 min. White arrowhead indicates SEPT6-GFP recruitment to the bacterial division site. Scale bar = 1 µm.

Figure S2. Cardiolipin Promotes Septin Recruitment to *Shigella* Membrane Curvature, Related to Figure 2.

(A) Diagram illustrating CL synthesis and localisation in *Shigella*. The three enzymes catalysing the formation of CL are highlighted in purple. CDP-DAG, CDP-diacylglycerol; PGP, phosphatidylglycerophosphate; PS, phosphatidylserine; PG, phosphatidylglycerol; PE, phosphatidylethanolamine; CL, cardiolipin. CL is synthesized in the inner membrane and gets transported to the outer membrane via PbgA (Dong et al., 2016).

(B) Membrane lipid strips were incubated with 200 nM SEPT9 like in Figure 3A.

(C). Negative ion mode MALDI-TOF MS spectra showing distinct cardiolipin peaks in *S. flexneri* WT (purple), which are lost in ΔCL.

(D) Growth of *S. flexneri* WT or ΔCL was determined by measuring the optical density (OD_{600}) every 30 min for 15 h using a microplate reader. Graph shows mean OD_{600} ± SEM from 3 independent experiments. Student’s t-test, ns $p > 0.05$.

(E) HeLa cells were infected with *S. flexneri* WT or ΔCL for 3 h 40 min. Graph represents mean % ± SEM of *S. flexneri* polymerising actin (including actin tails and clouds). Values from n = 4945 bacterial cells from 3 independent experiments. Student’s t-test, ns $p > 0.05$.

(F) HeLa cells were infected with *S. flexneri* ΔCL for 3 h 40 min, showing cardiolipin-deficient *Shigella* polymerise actin and are not entrapped in SEPT7 cages. Scale bar = 1 µm.

Figure S3. Septins Entrap Actively Dividing Bacteria, Related to Figure 3.

(A-C) Untreated (CTRL), erythromycin (EM) treated or trimethoprim (TMP) treated *S. flexneri* x-light GFP infected HeLa cells at 3 h 40 min post infection. Graph in (A) shows mean % ± SEM of metabolically active *S. flexneri* (i.e. expressing GFP) (Sirianni et al., 2016). Graph in (C) shows mean % ± SEM of actin-polymerising bacteria in CTRL,

EM- or TMP-treated cells. Values from n = 5215 bacterial cells from 3 independent experiments. Student's t-test, ns $p > 0.05$. Representative images showing *S. flexneri* recruiting actin (white arrowheads) (B). Scale bars = 5 µm.

(D) HeLa cells were infected with *S. flexneri* SulA for 3 h 40 min, FM4-64X was added 30 min prior to fixation and samples were stained for endogenous SEPT7. Inset image highlights SEPT7 cage-like structure around filamentous *S. flexneri*. Scale bar = 1 µm.

(E) SEPT6-GFP HeLa cells were infected with *S. flexneri* for 40 min, cephalexin was added for further 45 min and samples were imaged every 2 min. Video frames show septin recruitment to invaginated, filamentous bacteria before cephalexin can fully act.

Figure S4. Septin Cages Inhibit Bacterial Cell Division via Autophagy and Lysosome Fusion, Related to Figure 4.

(A) Whole-cell lysates of untreated (CTRL) or chloroquine treated (CQ) HeLa cells were immunoblotted for p62. GAPDH was used as a loading control.

(B) Untreated (CTRL) or chloroquine treated (CQ) HeLa cells were immunolabelled for p62 and stained for DAPI. Scale bars = 5 µm.

Figure S1. Septins Recognise Micron-Scale Bacterial Curvature.

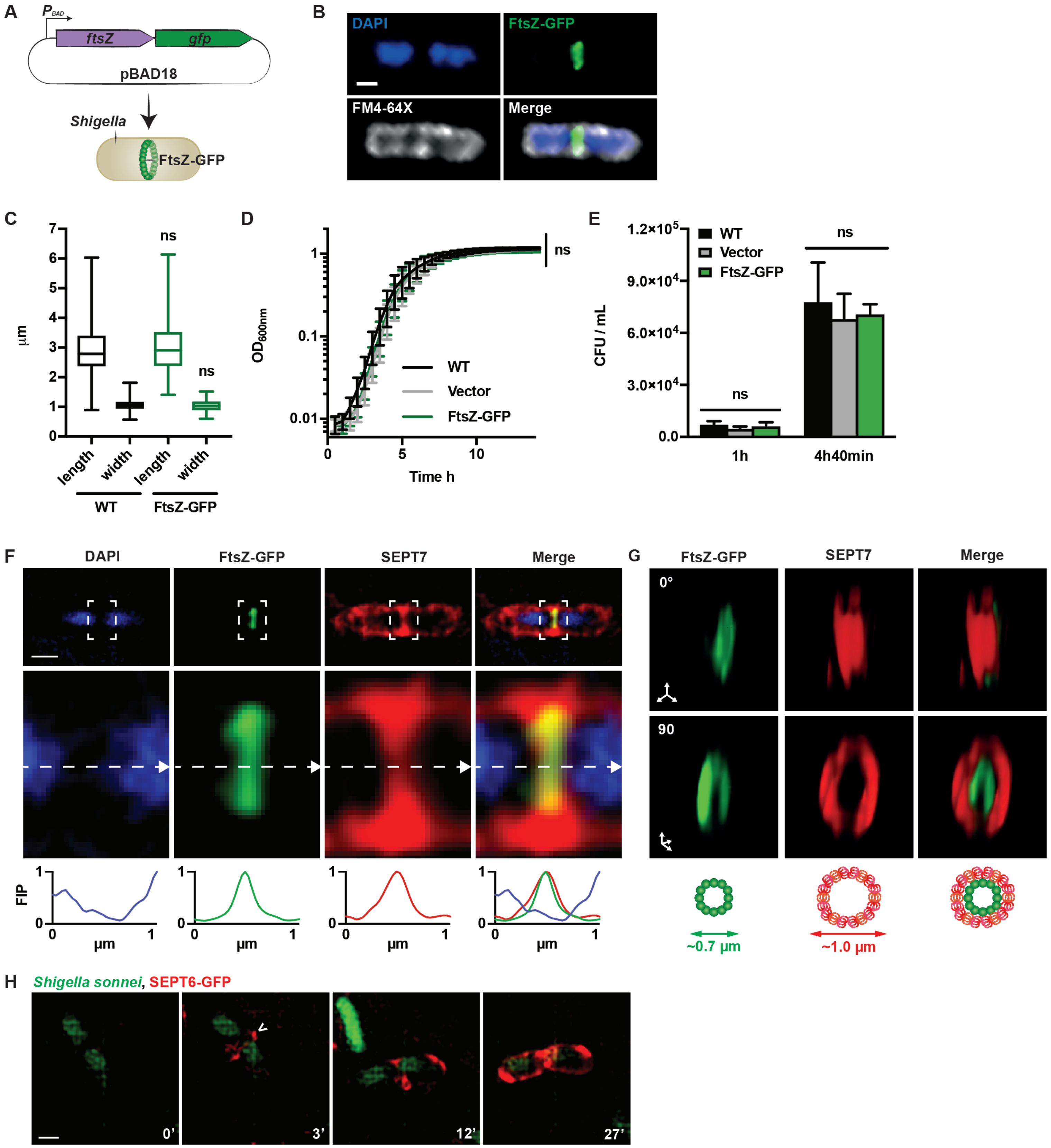

Figure S2. Cardiolipin Promotes Septin Recruitment to *Shigella* Membrane Curvature.

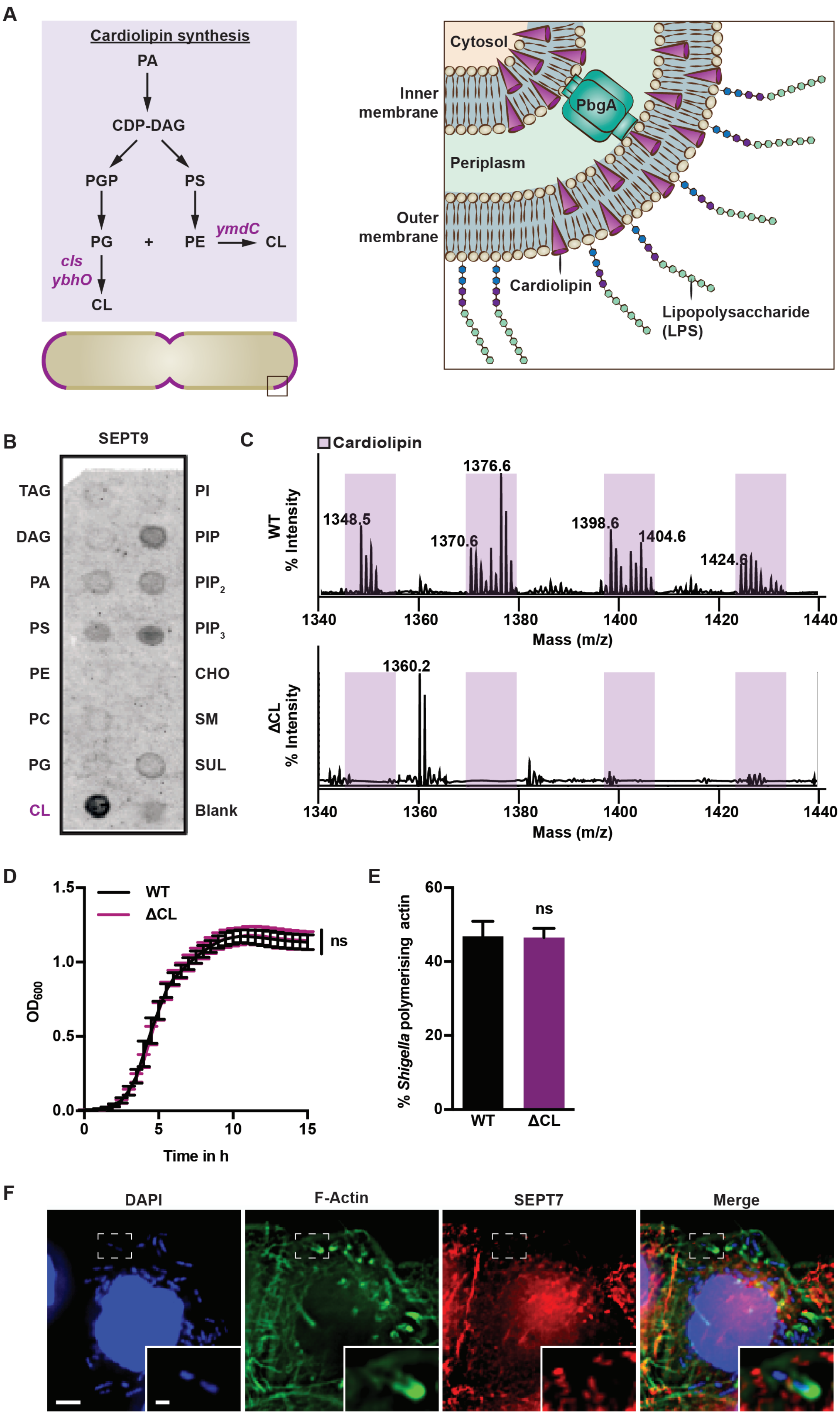

Figure S3. Septin Cages Entrap Actively Dividing Bacteria.

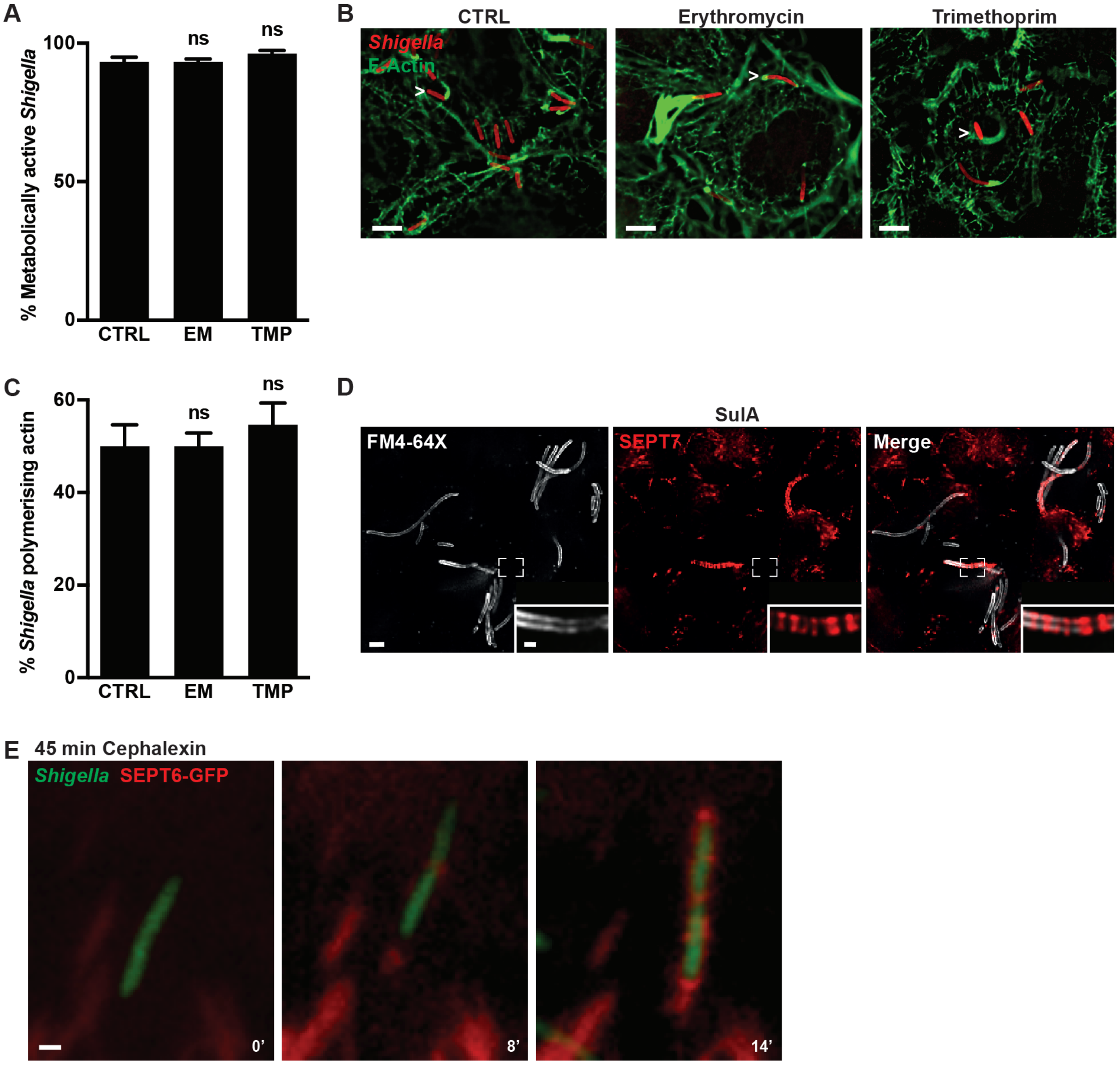

Figure S4. Septin Cages Inhibit Bacterial Cell Division via Autophagy and Lysosome Fusion.

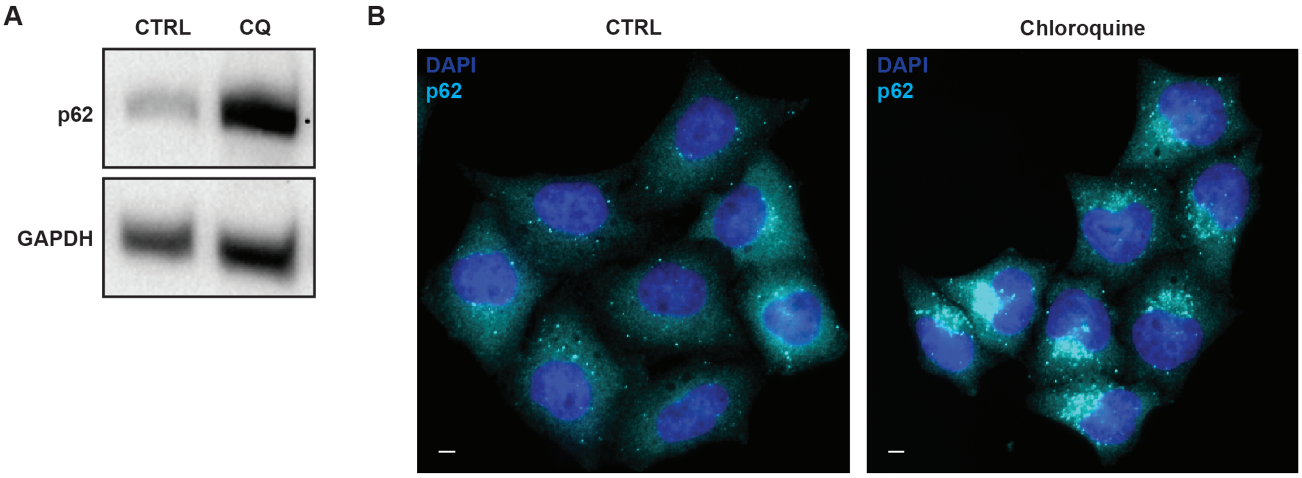

Table S1. Primers used in this study. Related to STAR Methods sections 'Construction of Deletion Mutants' and 'Plasmid construction'.

Primer	DNA sequence
SK-19	CCAGCATTCCTACGTAAGCAAGCTGATAGTAAAGGTGAAGAACTGTTCACCG
SK-20	CACCGGTGAACAGTTCTTCACCTTTACTATCAGCTTGCTTACGTAGGAATGC
SK-45	CTGGTCTCGAATTCATATGTTTGAACCAATGGAACTTACC
SK-54	GTATGTCGACGCTTATTTGTAGAGTTCATCCATGCCG
SK-101	GTATGTCGACGCACTTAATGATACAAATTAGAGTGAA
SK-102	CGATGAATTCATATGTACACTTCAGGCTATGC
SK-105	AGTCAGGCGATTGTTTAGATCCATATCCATAGTCACTACCTGTTTAACCTGTGTAG GCTGGAGCTGCTTC
SK-106	CCCCACTTCCGTTCTACTCCGCTTCATGTTAACTACTCTATGCAATAACACATATG AATATCCTCCTTAGT
SK-107	CGAGATTCAGTGACAAACTGAGCGGATCGAGATTACTGGACCCTACTGTCATGGG AATTAGCCATGGTCC
SK-108	ATGAAATGTAGCTGGCGCGAAGGCAATAAGATCCAGTTGCTGGAAAACGGTGTGT AGGCTGGAGCTGCTTC
SK-113	CACTGTGTTCGCAACATCCCGGTCAGTGTGGTCTTTTCCCTCTGGAGAAAGTGTA GGCTGGAGCTGCTTC
SK-114	TTACAATAACCATTCCACGGGCAATATCGACGCCAGTCTGACCATAACCCCATATG AATATCCTCCTTAGT
