# Supplementary material for: Septins Recognize and Entrap Dividing Bacterial Cells for Delivery to Lysosomes
Source: Cell Host Microbe. 2018 Dec 12;24(6):866–874.e4. doi: 10.1016/j.chom.2018.11.005 (PMC6299245; doi:10.1016/j.chom.2018.11.005)
Supplement: Document S2. Article plus Supplemental Information [file mmc5.pdf]

Short Article

Cell Host & Microbe

Septins Recognize and Entrap Dividing Bacterial Cells for Delivery to Lysosomes

Graphical Abstract

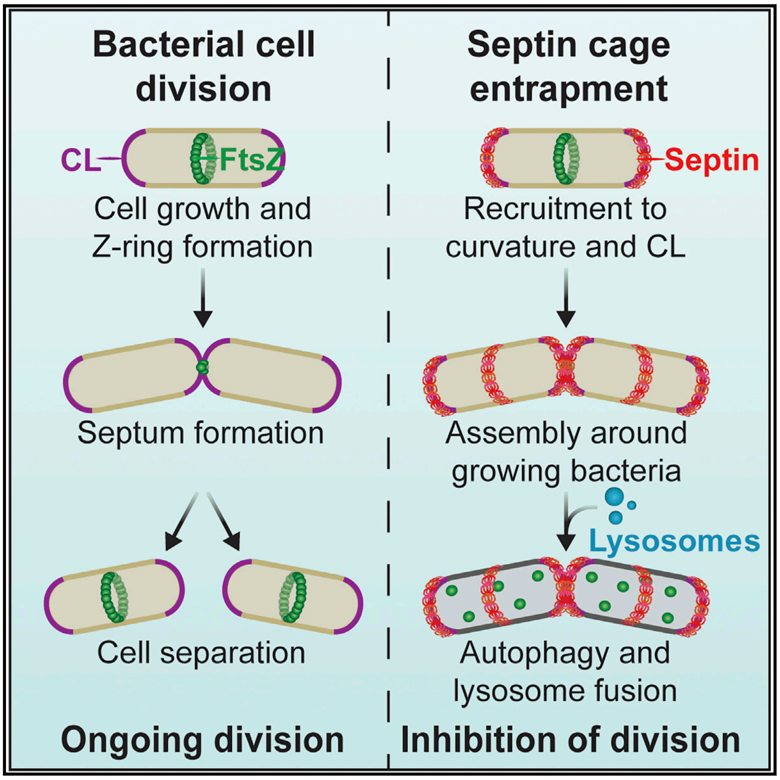

Authors

Sina Krokowski,
Damián Lobato-Márquez,
Arnaud Chastanet, ..., Elias T. Spiliotis,
Rut Carballido-López, Serge Mostowy

Correspondence

serge.mostowy@lshtm.ac.uk

In Brief

Septins are cytoskeleton components widely recognized for their role in eukaryotic cell division. Krokowski et al. discover that septins recognize dividing bacterial cells for entrapment and delivery to lysosomes. These results reveal a fundamental danger signal used by the host cell to recognize intracellular bacterial pathogens for cellular immunity.

Highlights

- Septins are recruited to micron-scale curvature of dividing intracellular bacteria
- Cardiolipin promotes the recruitment of septins to curved bacterial membranes
- Following recruitment, septins assemble into cages around growing bacterial cells
- Septins inhibit bacterial division via recruitment of autophagic and lysosomal machinery

Krokowski et al., 2018, Cell Host & Microbe *24*, 866–874
December 12, 2018 © 2018 The Author(s). Published by Elsevier Inc.
https://doi.org/10.1016/j.chom.2018.11.005

CellPress

CellPress

Cell Host & Microbe
Short Article

Septins Recognize and Entrap Dividing Bacterial Cells for Delivery to Lysosomes

Sina Krokowski,[1,2] Damián Lobato-Márquez,[1,2] Arnaud Chastanet,[3] Pedro Matos Pereira,[4] Dimitrios Angelis,[5] Dieter Galea,[1] Gerald Larrouy-Maumus,[6] Ricardo Henriques,[4] Elias T. Spiliotis,[5] Rut Carballido-López,[3] and Serge Mostowy[1,2,7,*]

[1]Section of Microbiology, MRC Centre for Molecular Bacteriology and Infection, Imperial College London, London SW7 2AZ, UK
[2]Department of Immunology & Infection, London School of Hygiene & Tropical Medicine, London WC1E 7HT, UK
[3]MICALIS, INRA, AgroParisTech, Université Paris-Saclay, Jouy-en-Josas 78350, France
[4]Quantitative Imaging and NanoBiophysics Group, MRC Laboratory for Molecular Cell Biology and Department of Cell and Developmental Biology, University College London, London WC1E 6BT, UK
[5]Department of Biology, Drexel University, Philadelphia, PA 19104, USA
[6]Faculty of Natural Sciences, Department of Life Sciences, MRC Centre for Molecular Bacteriology and Infection, Imperial College London, London SW7 2AZ, UK
[7]Lead Contact
*Correspondence: serge.mostowy@lshtm.ac.uk
https://doi.org/10.1016/j.chom.2018.11.005

SUMMARY

The cytoskeleton occupies a central role in cellular immunity by promoting bacterial sensing and antibacterial functions. Septins are cytoskeletal proteins implicated in various cellular processes, including cell division. Septins also assemble into cage-like structures that entrap cytosolic *Shigella*, yet how septins recognize bacteria is poorly understood. Here, we discover that septins are recruited to regions of micron-scale membrane curvature upon invasion and division by a variety of bacterial species. Cardiolipin, a curvature-specific phospholipid, promotes septin recruitment to highly curved membranes of *Shigella*, and bacterial mutants lacking cardiolipin exhibit less septin cage entrapment. Chemically inhibiting cell separation to prolong membrane curvature or reducing *Shigella* cell growth respectively increases and decreases septin cage formation. Once formed, septin cages inhibit *Shigella* cell division upon recruitment of autophagic and lysosomal machinery. Thus, recognition of dividing bacterial cells by the septin cytoskeleton is a powerful mechanism to restrict the proliferation of intracellular bacterial pathogens.

INTRODUCTION

Septins are highly conserved filament-forming proteins that play key roles in eukaryotic cell division (Mostowy and Cossart, 2012; Saarikangas and Barral, 2011). Originally discovered for their role in division of budding yeast (Hartwell, 1971), recent work has shown that septins also play a role in division of mitochondria (Pagliuso et al., 2016; Sirianni et al., 2016). In humans, the 13 septins are classified into four homology groups (SEPT2, SEPT3, SEPT6, and SEPT7) where septins from different groups form hetero-oligomers and assemble into non-polar filaments, which can form higher-order structures including bundles and rings (Mostowy and Cossart, 2012). Septins have recently been shown to recognize areas of the plasma membrane presenting micron-scale curvature, including the cytokinetic furrow and phagocytic cup (Bridges et al., 2016). Although viewed as a fundamental property of the septin cytoskeleton (Cannon et al., 2017), signals responsible for septin recruitment to membrane, and the precise role of septin-membrane interactions, remain poorly understood.

Shigella flexneri is taxonomically indistinguishable from *Escherichia coli*, but possesses a virulence plasmid encoding a type III secretion system (T3SS) that enables host cell invasion (Parsot, 2009). Following invasion, *Shigella* escapes from the phagosome to proliferate in the cytosol and polymerize actin tails for cell-to-cell spread (Welch and Way, 2013). To defend against *Shigella* invasion, host cells use a variety of mechanisms, including autophagy (Ogawa et al., 2005), guanylate-binding proteins (GBPs) (Li et al., 2017; Wandel et al., 2017), and septin-mediated cellular immunity (Mostowy et al., 2010). To prevent bacterial dissemination, septins entrap actin-polymerizing bacteria in ~1-μm (diameter) cage-like structures (Mostowy et al., 2010). It has been shown that ~50% of entrapped bacteria are metabolically inactive (Sirianni et al., 2016), but their fate is mostly unknown.

The eukaryotic cytoskeleton is well known to rearrange during infection and play a crucial role in host-microbe interactions (Haglund and Welch, 2011). Components of the cytoskeleton mediate cellular immunity by enabling bacterial detection and mobilizing antibacterial mechanisms (Mostowy and Shenoy, 2015). Despite the septin cage representing an important link between the cytoskeleton and cellular immunity, we lack fundamental insights into how septins recognize bacteria for cage entrapment. Here, we discover that septin recognition of membrane curvature and growth during bacterial cell division is an unsuspected mechanism used by the host cell to defend against invasive pathogens.

 © 2018 The Author(s). Published by Elsevier Inc.
This is an open access article under the CC BY license (http://creativecommons.org/licenses/by/4.0/).

RESULTS

Septins Recognize Micron-Scale Bacterial Curvature

How do septins recognize bacteria for entrapment? Considering that septins sense micron-scale curvature of eukaryotic membrane (Bridges et al., 2016), we hypothesized that septins are recruited to *Shigella* (cells ~1 μm in diameter) in a curvature-dependent manner. To test this, we examined the recruitment of SEPT6-GFP to *Shigella* M90T mCherry using time-lapse microscopy. We observed that for 87.4% ± 1.9% of entrapped bacteria, septins are first recruited to the division site and/or the cell poles (both displaying high curvature) before they assemble into cage-like structures (Figures 1A and 1B; Video S1), suggesting a role for bacterial curvature in septin recruitment.

Bacterial invagination at the division site is driven by the bacterial tubulin homolog FtsZ, which forms the cytokinetic Z-ring. To follow the division site of intracellular bacteria, we expressed an inducible *ftsZ-gfp* fusion in *S. flexneri* (Figures S1A–S1E). Strikingly, time-lapse microscopy of SEPT6-RFP HeLa cells infected with *Shigella* FtsZ-GFP revealed that SEPT6-RFP can overlap with the Z-ring for up to 36 min (Figures 1C and 1D). Fixed microscopy of 147 *Shigella*-septin cages showed that SEPT7 overlaps with FtsZ-GFP at the bacterial division site for 68.5% ± 3.7% of Z-ring-positive bacteria (Figure 1E), and structural illumination microscopy (SIM) confirmed that SEPT7 alignment to FtsZ-GFP is highly conserved (Figures S1F and S1G). To test whether septins align with the Z-ring due to membrane invagination, we simultaneously labeled the Z-ring and bacterial membrane and performed quantitative microscopy. Here, we observed that septin alignment to the Z-ring is significantly increased (3.0 ± 0.4-fold) when membrane is invaginated, as compared with when membrane is not invaginated (Figure 1F). These data strongly suggest that bacterial curvature generated by Z-ring constriction promotes septin localization to the bacterial division site.

We questioned whether septins can be recruited to other bacteria presenting micron-scale curvature. *Shigella sonnei* is closely related to *S. flexneri* and similarly entrapped in septin cages. Using time-lapse SIM of HeLa SEPT6-GFP cells infected with *S. sonnei* mCherry, we observed initial septin recruitment to bacterial division sites and/or cell poles (Figure S1H). We next infected HeLa cells with invasive *Pseudomonas aeruginosa* PAK-GFP and discovered by quantitative microscopy that 17.5% ± 1.4% of intracellular bacteria are entrapped in SEPT7 cage-like structures at 4 hr post infection (Figure 1G). Time-lapse microscopy of SEPT6-RFP HeLa cells infected with *P. aeruginosa* GFP showed initial septin recruitment to highly curved bacterial membrane (Figure 1H; Video S2). To investigate whether septins can also recognize Gram-positive bacteria, we infected SEPT6-RFP HeLa cells with invasive *Staphylococcus aureus* RN6390 GFP, and time-lapse microscopy showed septin recruitment to *S. aureus* division sites (Figure 1I; Video S3). Together, these results highlight septin recruitment to micron-scale curvature presented by a variety of invasive bacterial species.

Cardiolipin Promotes Septin Recruitment to *Shigella* Membrane Curvature

Considering that septins bind anionic phospholipids of eukaryotic membranes, we reasoned that septins might also bind anionic phospholipids specific to bacterial curvature. In rod-shaped bacteria, the lipid species cardiolipin (CL) and phosphatidylglycerol (PG) are enriched at the bacterial division site and cell poles (Kawai et al., 2004; Oliver et al., 2014) (Figure S2A). We purified SEPT2/6/7 and SEPT9 and tested for lipid binding *in vitro* using membrane lipid strips containing 15 different phospholipids (Figures 2A and S2B). SEPT2/6/7 and SEPT9 bound strongly to CL but not significantly to PG. We also tested for CL binding of SEPT2, SEPT6, SEPT9, and SEPT6/7 using dot-blot assays with purified CL from *E. coli* (Figure 2B). Together, these results show that SEPT2 and SEPT6 do not bind CL whereas SEPT2/6/7, SEPT6/7, and SEPT9 do.

To confirm that septins bind bacterial membrane, we performed liposome flotation assays using purified SEPT2/6/7 and total lipid extracts from *S. flexneri*. Septins clearly bound vesicles made from bacterial total lipid extracts (Figure 2C). We then constructed a *S. flexneri* Δ*cls*Δ*ymdC*Δ*ybhO* (ΔCL) mutant strain void of CL (Figure S2C) and tested for SEPT2/6/7 binding. In this case, we found that SEPT2/6/7 binds significantly more (6.3 ± 3.5-fold) to vesicles produced from wild-type (WT) *Shigella* than ΔCL *Shigella* (Figure 2C). Consistent with this, quantitative microscopy revealed that ΔCL *Shigella* are significantly less (1.4 ± 0.1-fold) entrapped in SEPT7 cages than WT *Shigella* (Figures 2D and S2D–S2F).

Is septin interaction with CL a recruitment mechanism or can it additionally promote cage assembly? To address this, we performed time-lapse microscopy of SEPT6-GFP HeLa cells infected with WT or ΔCL *Shigella* mCherry. We observed that septin recruitment to bacterial curvature lacking CL is significantly decreased (2.9 ± 0.3-fold) compared with bacterial curvature presenting CL (n = 276 WT versus n = 93 ΔCL total bacterial cells). To further disentangle the role of curvature and CL in septin recruitment, we prepared supported bacterial lipid bilayers on 1- or 5-μm (diameter) beads, and compared septin recruitment here with beads coated with *S. flexneri* lipids presenting CL or not. We found that septins preferentially associate with 1-μm beads and that CL promotes septin recruitment to both 1- and 5-μm beads (Figures 2E and 2F). From these results, we conclude that CL can promote the recruitment of septins to bacterial curvature.

Septin Cages Entrap Actively Dividing Bacteria

To gain insights into bacterial factors required for cage entrapment to proceed after septin recruitment, we used antibiotics targeting different processes of bacterial cell division. First, we inhibited bacterial cell growth by treating infected cells with erythromycin or trimethoprim, antibiotics that interfere with bacterial protein synthesis or DNA replication, respectively. Under these conditions SEPT7 cages are rarely observed (Figure 3A), despite bacteria being metabolically active and recruiting actin similar to untreated bacteria (Figures S3A–S3C). Time-lapse microscopy showed that septins are transiently recruited to the poles of trimethoprim-treated bacteria, but mostly (i.e., for 79.3% ± 2.6% of bacteria) fail to assemble into cages (Figure 3C). Together, these data support a model in which bacterial curvature is required for septin recruitment and bacterial growth is required for septin cage entrapment.

To further test our model, we treated infected cells with cephalexin, an antibiotic that inhibits cell separation and thus promotes filamentation (Pogliano et al., 1997). In this case, the

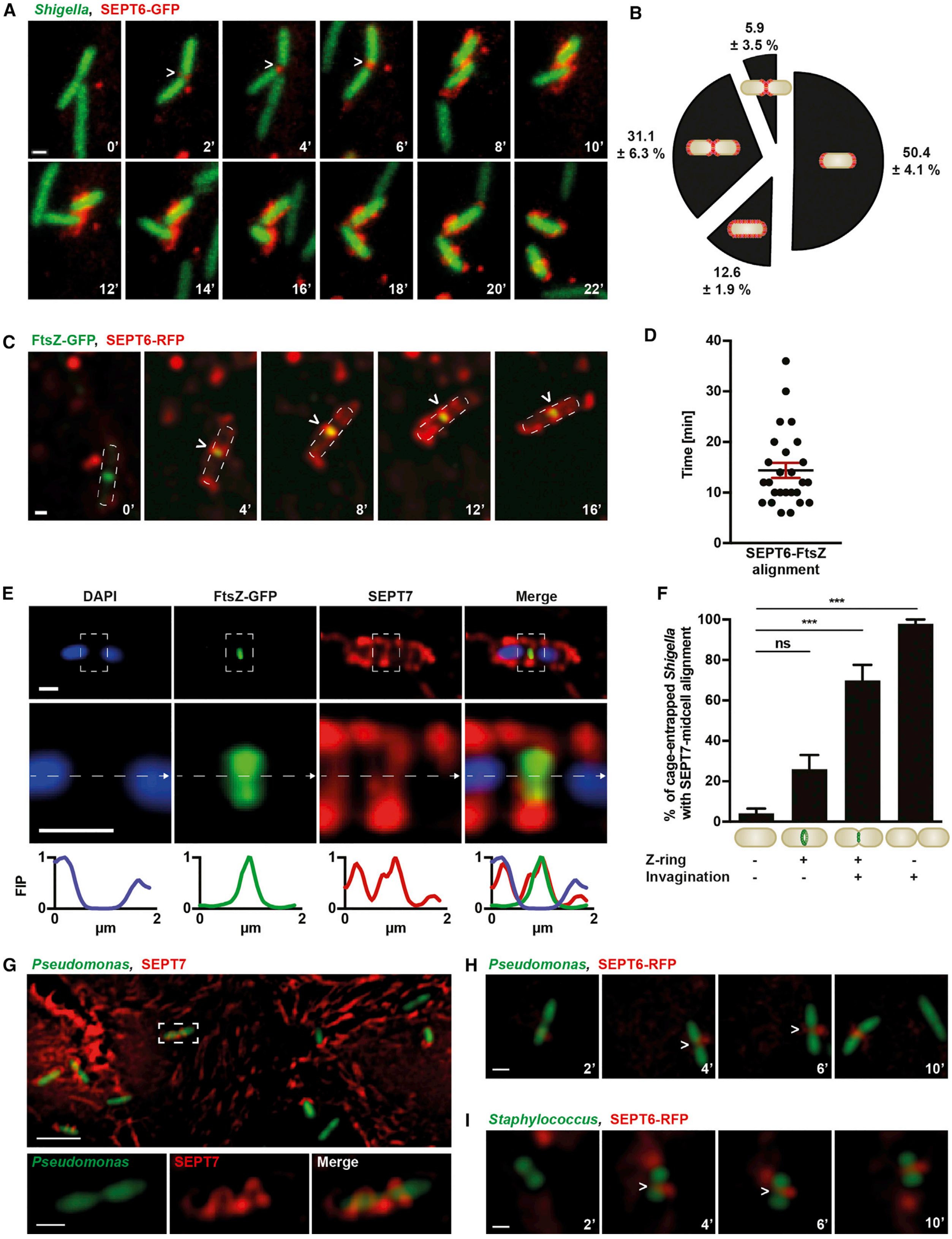

(legend on next page)

percentage of *Shigella* entrapped in SEPT7 cage-like structures is significantly increased (1.9 ± 0.1-fold) compared with untreated bacteria (Figures 3D and 3E). Similar results were obtained when testing septin caging of filamentous bacteria created by overproducing the FtsZ inhibitor SulA (Figures 3F and S3D), which in contrast to cephalexin inhibits cell separation before Z-ring formation. We hypothesized that septins may be recruited to CL-rich curved cell poles of filamentous bacteria. To test this, we infected SEPT6-GFP HeLa cells with *Shigella* mCherry, treated them with cephalexin for 3 hr, and performed time-lapse microscopy. However, we could rarely see initial septin recruitment to filamentous bacteria, suggesting that septin recruitment happens to membrane curvature prolonged by cephalexin before it inhibits cell separation. To confirm this, we treated infected cells with cephalexin but investigated septin recruitment before the drug could fully act (45 min after drug addition). Here, we clearly observed septin recruitment to dividing bacteria before cells could grow into filaments (Figure S3E). Collectively, pharmacologic and genetic manipulation of *Shigella* reveals that, following septin recruitment, bacterial cell growth is required for septin cage entrapment.

Septin Cages Inhibit Bacterial Cell Division via Autophagy and Lysosome Fusion

To address the impact of septin caging on bacterial cell division, we tested whether septin cages inhibit the division of entrapped *Shigella*. We used time-lapse microscopy to focus on the fate of Z-ring-positive bacteria following septin cage recruitment. We observed that 61.3% ± 4.0% of septin cage-entrapped *Shigella* lose their Z-ring (Figures 4A and 4B), and that Z-ring disassembly takes 27.8 ± 3.9 min following septin cage recruitment (Figure 4C). In agreement with a role for septin caging in the inhibition of bacterial cell division, time-lapse microscopy showed that, remarkably, 92.7% ± 2.5% of bacteria fail to divide following septin cage entrapment.

To test the role of autophagy in the inhibition of bacterial cell division by septin cages, we quantified Z-ring-positive *Shigella* recruiting septins and p62 (early autophagy marker) or LC3B (early and late autophagy marker). We found that *Shigella* recruiting both septins and p62 are significantly less (1.9 ± 0.2-fold) Z-ring positive, as compared with *Shigella* which recruit septins but not p62 (Figures 4D and 4E). Moreover, *Shigella* are significantly less (1.5 ± 0.1-fold) Z-ring positive when recruiting both SEPT7 and p62, as compared with bacteria recruiting p62 alone. Similar results are obtained using LC3B (Figures 4F and 4G). Together, these data suggest that septins are necessary but not sufficient for *Shigella* Z-ring disassembly, and highlight an interdependence between septins and autophagy in the inhibition of bacterial cell division.

Considering that septins are implicated in the fusion of lysosomes with endocytic membranes (Dolat and Spiliotis, 2016), we hypothesized that septin caging may promote the fusion of lysosomes with entrapped *Shigella*. In line with this, time-lapse microscopy captured 42.0% ± 5.8% of *Shigella*-SEPT6 cages becoming LysoTracker positive within the imaging period (Figure 4H). To test whether the antibacterial activity of septin cages is dependent on lysosome fusion, we treated infected cells with chloroquine to neutralize lysosomes and inhibit autophagy (Figures S4A and S4B). When infected cells are treated with chloroquine, entrapped *Shigella* fail to disassemble their Z-ring (Figures 4I and 4J). Collectively, these results demonstrate that septin cages entrap actively dividing bacteria and prevent further division events by autophagy and lysosome fusion.

DISCUSSION

How septins recognize intracellular bacteria for cage entrapment has remained unknown. Using single-cell analysis we found that septins are recruited to micron-scale curvature presented by *Shigella*, *Pseudomonas*, and *Staphylococcus*. In the case of *Shigella*, we show that septin recruitment is promoted by CL, which is enriched at such regions of high membrane curvature. Following recruitment, septins assemble into cages around actively growing *Shigella* to inhibit cell division via autophagy and lysosome fusion. These results uncover a mechanism used by the host cell to sense and restrict bacterial cell division, and have important implications for septin biology and cellular immunity.

Investigation of the host cell cytoskeleton during bacterial infection has enabled major discoveries in both infection and cell biology (Haglund and Welch, 2011; Welch and Way, 2013).

Figure 1. Septins Recognize Micron-Scale Bacterial Curvature

(A) Time-lapse of *S. flexneri* mCherry-infected SEPT6-GFP HeLa at 2 hr 10 min post infection imaged every 2 min. White arrowheads indicate septin recruitment to the bacterial division site. Scale bar, 1 μm. See also Video S1.

(B) Quantification of (A). The graph represents mean % ± SEM of septin recruitment to highly curved membrane areas (bacterial cell poles and/or bacterial midcell). Values from n = 79 bacterial cells from 8 independent experiments.

(C) Time-lapse of *S. flexneri* FtsZ-GFP-infected SEPT6-RFP HeLa cells at 2 hr 10 min imaged every 2 min. White arrowheads indicate SEPT6-RFP overlap with FtsZ-GFP at the bacterial division site. Dashed lines indicate bacterial contour. Scale bar, 1 μm.

(D) Quantification of (C). Graph shows individual times of SEPT6-FtsZ overlap from n = 26 bacterial cells including mean ± SEM from 6 independent experiments.

(E) Representative SEPT7 cage in *S. flexneri* FtsZ-GFP-infected HeLa cells at 3 hr 40 min post infection. Scale bars, 1 μm. Inset images highlight a septin ring at the bacterial division site. Fluorescent intensity profile (FIP) was taken of the dotted line along the midline of the cell in the inset image and normalized from 0 to 1.

(F) HeLa cells were infected for 3 hr 40 min with *S. flexneri* FtsZ-GFP and bacterial membrane was labeled with FM4-64X. Graph represents mean % ± SEM of SEPT7 aligning at midcell of entrapped *S. flexneri* when bacteria are either Z-ring negative and not invaginated (−, −), Z-ring positive and not invaginated (+, −), Z-ring positive and invaginated (+, +), or Z-ring negative after cell separation (−, +). Values from n = 289 bacterial cells from 3 independent experiments. One-way ANOVA; ns, $p > 0.05$; ***$p < 0.001$.

(G) *P. aeruginosa* GFP-infected HeLa cells at 4 hr post infection immunostained for SEPT7. Scale bars, 5 μm (main image) and 1 μm (inset).

(H) Time-lapse of *P. aeruginosa* GFP-infected SEPT6-RFP HeLa cells at 1 hr post infection imaged every 2 min. Scale bar, 1 μm. See also Video S2.

(I) Time-lapse of *S. aureus* GFP-infected SEPT6-RFP HeLa cells at 1 hr post infection imaged every 2 min. Scale bar, 1 μm. See also Video S3.

See also Figure S1.

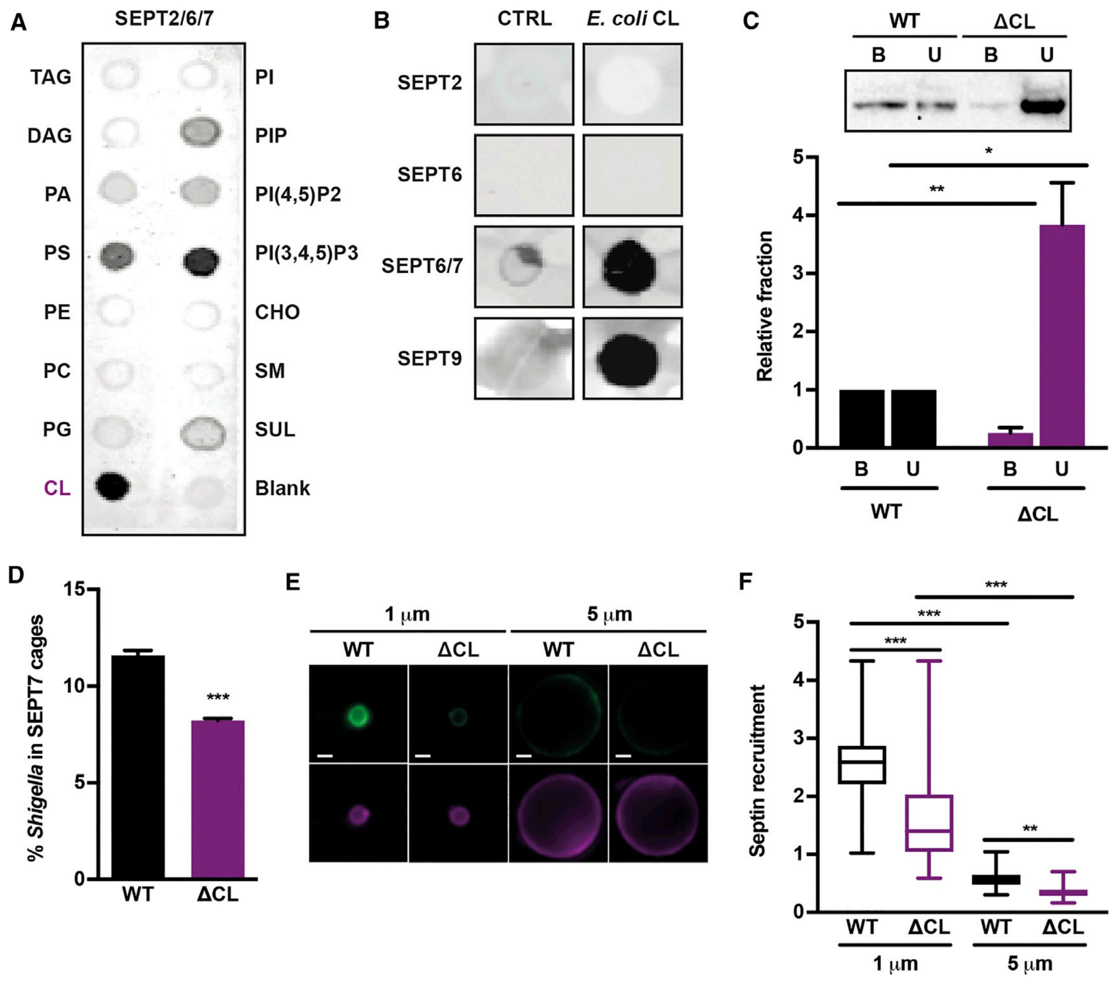

Figure 2. Cardiolipin Promotes Septin Recruitment to *Shigella* Membrane Curvature

(A) Membrane lipid strips were incubated with 200 nM SEPT2/6/7. TAG, triacylglycerol; DAG, diacylglycerol; PA, phosphatidic acid; PS, phosphatidylserine; PE, phosphatidylethanolamine; PC, phosphatidylcholine; PG, phosphatidylglycerol; CL, cardiolipin; PI, phosphatidylinositol; PIP, phosphatidylinositol phosphate; PI(4,5)P2, phosphatidylinositol bisphosphate; PI(3,4,5)P3, phosphatidylinositol trisphosphate; CHO, cholesterol; SM, sphingomyelin; SUL, sulfatide.

(B) Representative lipid dot blot in which 200 nM SEPT2, SEPT6 (negative control because no lipid interaction [Spiliotis, 2018]), SEPT6/7, or SEPT9 was incubated with a nitrocellulose membrane containing water (CTRL) or 10 nmol purified CL from *E. coli* (*E. coli* CL).

(C) SEPT2/6/7 was incubated with liposomes made from *S. flexneri* wild-type (WT) or ΔCL and centrifuged on a sucrose gradient. Representative western blot (top) shows equal volumes for the top fraction (B, liposome-bound SEPT2/6/7) and bottom fraction (U, unbound SEPT2/6/7). Bar graph shows mean ± SEM of the amount of SEPT2/6/7 in the bound (B) and unbound (U) fraction relative to liposomes made from WT from 3 independent experiments. Student's t test, $^{**}p < 0.01$, $^{*}p < 0.05$.

(D) Graph represents mean % ± SEM of SEPT7 cage-entrapped *S. flexneri* WT or ΔCL at 3 hr 40 min post infection. Values from n = 4,945 bacterial cells from 3 independent experiments. Student's t test, $^{***}p < 0.001$.

(E) Representative image of 50 nM SEPT2/6/7 labeled with NHS-Alexa Fluor 488 (green) recruitment to 1-μm or 5-μm silica beads coated in lipid bilayer from *S. flexneri* WT or ΔCL containing RhPE (purple). Scale bar, 1 μm.

(F) Quantification of (E). Graph shows median and whiskers (min to max) from $n \geq 243$ beads for each condition from 3 independent experiments. Kruskal-Wallis test, $^{***}p < 0.001$, $^{**}p < 0.01$.

See also Figure S2.

Although first identified in 1991 (Bi and Lutkenhaus, 1991) and studied extensively for more than 15 years, FtsZ had never been examined in pathogenic bacteria during infection of host cells. For the complete understanding of infectious processes, it will be crucial to study FtsZ and other components of the bacterial cytoskeleton, including the actin homolog MreB and septin-like proteins MinCD, during infection. Here, we visualize FtsZ during infection and reveal that septins recognize actively dividing bacterial cells. Considering this, one should expect that septins are less efficient in recognition of non-dividing bacterial cells and sensitive to bacteria changing morphology during infection. It will thus be of great interest to investigate a role for septins in selection for bacterial pathogens that can escape detection by cellular immunity and remain dormant in host cells.

CL has recently been linked to bacterial virulence. In the case of *Salmonella* Typhimurium, CL delivery to the outer membrane

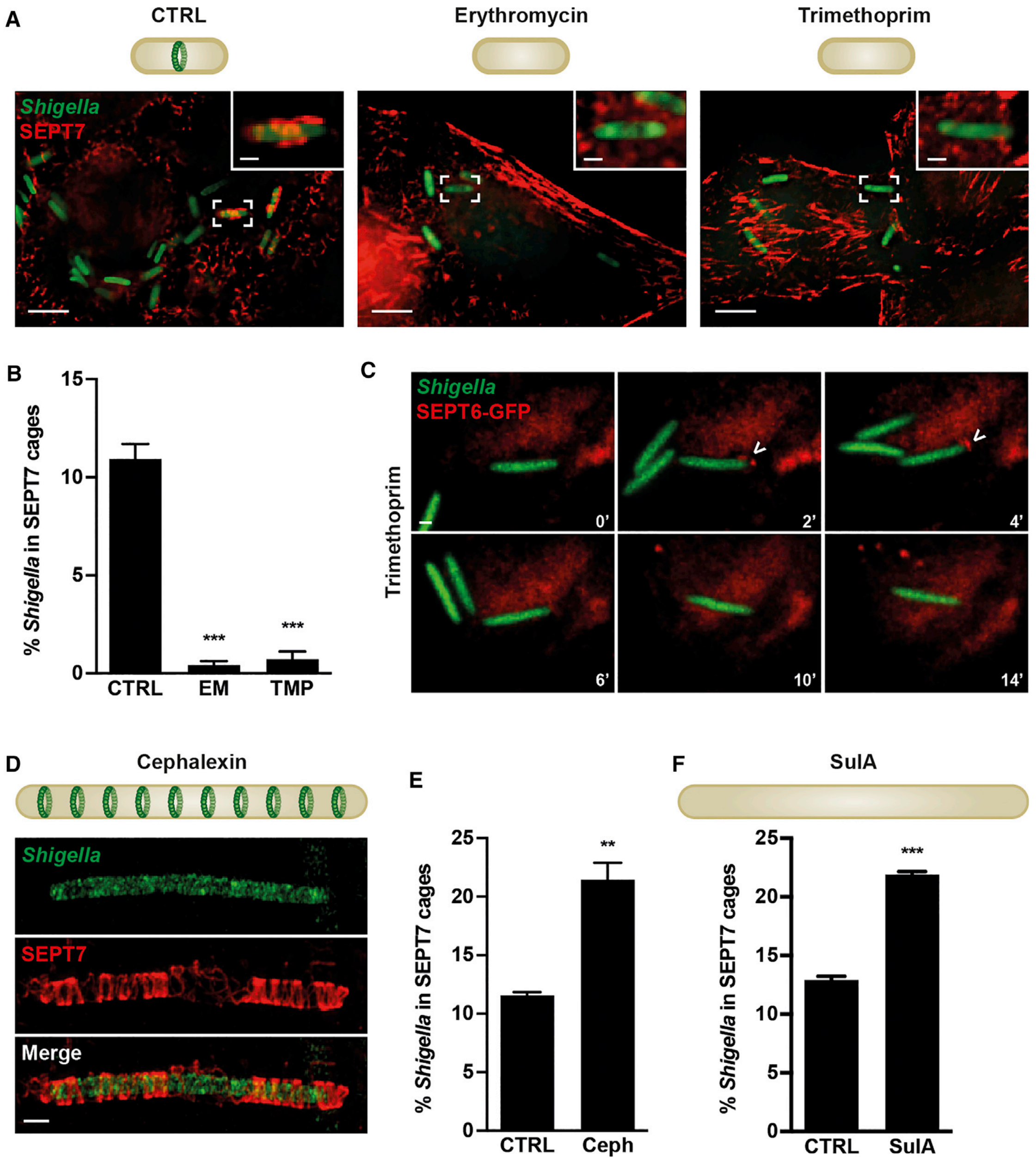

Figure 3. Septin Cages Entrap Actively Dividing Bacteria

(A) Diagrams illustrate that untreated bacteria (CTRL) assemble a Z-ring at bacterial midcell and erythromycin (EM) and trimethoprim (TMP) inhibit bacterial cell division and Z-ring assembly. Untreated (CTRL), EM-treated, or TMP-treated *S. flexneri* x-light GFP-infected HeLa cells at 3 hr 40 min post infection. Scale bars, 5 μm (main image) and 1 μm (inset).

(B) Quantification of (A). Graph shows mean % ± SEM of *S. flexneri* entrapped in SEPT7 cages in CTRL, EM-treated, or TMP-treated cells. Values from n = 5,215 bacterial cells from 3 independent experiments. Student's t test, ***p < 0.001.

(C) HeLa SEPT6-GFP cells were infected with *Shigella* mCherry, treated with TMP for 2 hr, and imaged every 2 min. Video frames (representative for n = 76 bacterial cells from 3 independent experiments) show temporary septin recruitment to the pole but no assembly into septin cages. Scale bar, 1 μm.

(D) Diagram illustrates cephalexin-treated bacterium, which elongates and forms division sites (i.e., FtsZ positive). SIM image of SEPT7 cage around cephalexin-treated *S. flexneri* GFP at 3 hr 40 min inside HeLa cell. Scale bar, 1 μm.

(E) Graph represents mean % ± SEM of *S. flexneri* entrapped in SEPT7 cage-like structures in untreated (CTRL) or cephalexin-treated (Ceph) cells. Values from n = 2,487 bacterial cells for CTRL and n = 499 bacterial cells from Ceph samples from 3 independent experiments. Student's t test, **p < 0.01.

(F) Diagram illustrates that overproduction of SulA inhibits Z-ring formation leading to cell filamentation. Graph shows mean % ± SEM of *S. flexneri* entrapped in SEPT7 cage-like structures in untreated (CTRL) or SulA-overproduced (SulA) samples. Values from n = 3,693 bacterial cells from 3 independent experiments. Student's t test, ***p < 0.001.

See also Figure S3.

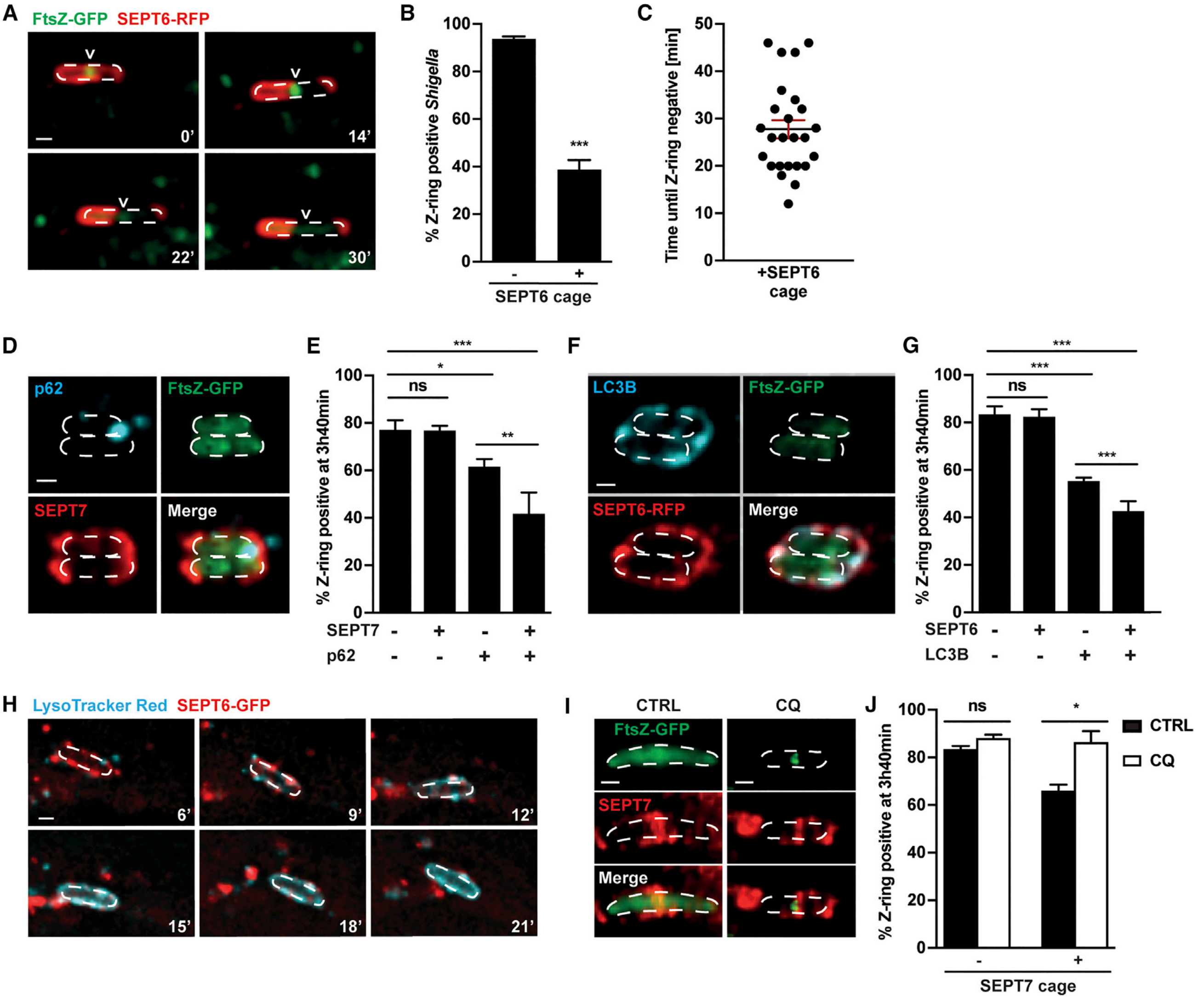

Figure 4. Septin Cages Inhibit Bacterial Cell Division via Autophagy and Lysosome Fusion

(A) Time-lapse of *S. flexneri* FtsZ-GFP-infected SEPT6-RFP HeLa cells at 2 hr 10 min post infection imaged every 4 min showing late stages of septin cage. Arrowheads point to the progressive disassembly of the Z-ring inside the bacterium (dashed outline). Scale bar, 1 μm.

(B) Quantification of (A). Graph shows mean % ± SEM of non-entrapped *S. flexneri* (-SEPT6 cage) or septin cage-entrapped *S. flexneri* (+SEPT6 cage) becoming Z-ring negative during imaging. Values from n = 1,721 bacterial cells from 8 independent experiments. Only bacteria that were entrapped for at least 3 consecutive time frames were considered as SEPT6 cage entrapped. Student's t test, ***$p < 0.001$.

(C) Quantification of (A). Graph shows the time from SEPT6 cage entrapment until Z-ring disassembly including mean ± SEM from n = 25 bacterial cells from 6 independent experiments. Only videos starting with septin recruitment and ending with Z-ring disassembly were considered for this quantification.

(D–G) *S. flexneri* FtsZ-GFP-infected HeLa cells at 3 hr 40 min post infection. Image shows two SEPT7 (D) or SEPT6 (F) cages positive for p62 (D) or LC3B (F) entrapping Z ring negative bacteria. Scale bar, 1 μm. Graphs show mean % ± SEM of Z-ring-positive bacteria (E and G). Values from n = 3,292 (E) or n = 1,737 (G) bacterial cells from 4 independent experiments Student's t test, ns, $p > 0.5$; ***$p < 0.001$.

(H) Time-lapse of *S. flexneri*-infected, LysoTracker red-labeled SEPT6-GFP HeLa cells at 2 hr 10 min imaged every 3 min. Frames show late stages of septin cage-entrapped *Shigella* (dashed outline). Scale bar, 1 μm.

(I) SEPT7 cages in *S. flexneri* FtsZ-GFP-infected untreated (CTRL) or chloroquine-treated (CQ) HeLa cells at 3 hr 40 min. Scale bar, 1 μm.

(J) Quantification of (I). Graph shows mean % ± SEM of Z-ring-positive *S. flexneri* outside septin cages (−SEPT7 cage) and inside septin cages (+SEPT7 cage) in CTRL or CQ-treated cells. Values from n = 5,047 bacterial cells from 3 independent experiments. Student's t test, ns, $p > 0.5$; *$p < 0.05$.

See also Figure S4.

can promote membrane barrier function and intracellular survival (Dalebroux et al., 2015). In the case of *S. flexneri*, CL promotes IcsA localization on the bacterial surface for actin-based motility (Rossi et al., 2017). What is the precise role of CL in septin recruitment? Our results suggest that septins bind CL for recruitment to highly curved bacterial membrane. How phospholipids and membrane curvature recruit septins in eukaryotic cells is not fully known (Cannon et al., 2017). We thus propose that

in-depth investigation of the *Shigella*-septin cage will help to describe the coordination between lipids, membrane curvature, and septin recruitment. Other factors besides CL binding and curvature may contribute to the recruitment of septins to intracellular *Shigella*. To discover this, future work will be required to screen bacterial surface components that can influence septin recruitment.

Cellular effector mechanisms that recognize invasive bacterial pathogens are the subject of intense investigation (Kieser and Kagan, 2017; Randow et al., 2013). It has recently become clear that the cytoskeleton is a key component of cellular immunity (Mostowy and Shenoy, 2015). However, the full breadth of cytoskeletal roles in host defense remains to be established. Our data reveal that septins recognize bacterial cell division for host defense. Here, we focus on *S. flexneri*, a World Health Organization priority pathogen also used as a valuable tool to investigate factors crucial for cellular immunity including autophagy, neutrophil extracellular traps, nucleotide-binding oligomerization domain-like receptors, and GBPs. Considering that septin recruitment has been described for a wide variety of bacterial pathogens (reviewed in Torraca and Mostowy, 2016) and vaccinia virus (Pfanzelter et al., 2018), it will next be important to test how widespread is the role of cell shape and membrane curvature in pathogen sensing.

Septins were discovered for their role in yeast cell division (Hartwell, 1971). Recently, septins have received great attention for their role in mitochondrial division (Pagliuso et al., 2016; Sirianni et al., 2016). In this study, we discover that septins also recognize bacterial cell division to restrict the proliferation of invasive pathogens. Having identified a link between cell division and cellular immunity, our results reveal a fundamental danger signal used by the host cell to recognize intracellular bacteria. Future work will determine how septin biology can be harnessed for therapeutic purposes, including control of antibiotic-resistant infections.

STAR★METHODS

Detailed methods are provided in the online version of this paper and include the following:

- KEY RESOURCES TABLE
- CONTACT FOR REAGENT AND RESOURCE SHARING
- EXPERIMENTAL MODEL AND SUBJECT DETAILS
  - Bacterial Strains
  - Cell Lines
- METHOD DETAILS
  - Bacterial Growth
  - Construction of Deletion Mutants
  - Plasmid Construction
  - Cardiolipin Detection Using MALDI-TOF MS
  - Protein-Lipid Overlay Assays
  - Liposome Flotation Assay
  - Septin Recruitment to Supported Lipid Bilayer Microspheres
  - Antibodies
  - Bacterial Infection of Epithelial Cells
  - Fluorescence Microscopy of Infected Cells
  - Structured Illumination Microscopy
- QUANTIFICATION AND STATISTICAL ANALYSIS

SUPPLEMENTAL INFORMATION

Supplemental Information includes four figures, one table, and three videos and can be found with this article online at https://doi.org/10.1016/j.chom.2018.11.005.

ACKNOWLEDGMENTS

We thank S. Murdoch and T. Palmer for *S. aureus* RN6390-GFP and A. Filloux for *P. aeruginosa* PAK::tn7gfp. We thank C. Parsot, A. Clements, C. Cornilleau, and F. Caudron for helpful tools and discussion. We thank the Company of Biologists for Travel fellowship to S.K. D.L.-M. is funded by the European Union's Horizon 2020 research and innovation program under the Marie Skłodowska-Curie grant agreement no. H2020-MSCA-IF-2016-752022. P.M.P. and R.H. are funded by grants from the UK Biotechnology and Biological Sciences Research Council (BB/M022374/1; BB/P027431/1; BB/R000697/1), the UK Medical Research Council (MR/K015826/1), and Wellcome Trust (203276/Z/16/Z). D.A. and E.T.S. are funded by National Institutes of Health/National Institute of General Medical Sciences (GM097664). Work in the R.C.-L. laboratory was supported by a starting grant from the European Research Council (ERC-StG-311231) and an international grant from the French National Research Agency (ANR-12-ISV3-0004-01). Work in the S.M. laboratory is supported by a Wellcome Trust Senior Research Fellowship (206444/Z/17/Z), Wellcome Trust Research Career Development Fellowship (WT097411MA), and the Lister Institute of Preventive Medicine.

AUTHOR CONTRIBUTIONS

S.K., D.L.-M., A.C., P.M.P., D.A., D.G., and G.L.-M. conducted experiments. S.K., D.L.-M., A.C., P.M.P., R.H., E.T.S., R.C.-L., and S.M. designed experiments and analyzed results. S.K. and S.M. wrote the manuscript, and all authors commented on the manuscript.

DECLARATION OF INTERESTS

The authors declare that they have no conflict of interest.

Received: February 1, 2018
Revised: August 14, 2018
Accepted: November 5, 2018
Published: December 12, 2018

REFERENCES

Bai, X., Bowen, J.R., Knox, T.K., Zhou, K., Pendziwiat, M., Kuhlenbäumer, G., Sindelar, C.V., and Spiliotis, E.T. (2013). Novel septin 9 repeat motifs altered in neuralgic amyotrophy bind and bundle microtubules. J. Cell Biol. *203*, 895–905.

Bi, E., and Lutkenhaus, J. (1991). FtsZ ring structure associated with division in *Escherichia coli*. Nature *354*, 161–164.

Bridges, A.A., Jentzsch, M.S., Oakes, P.W., Occhipinti, P., and Gladfelter, A.S. (2016). Micron-scale plasma membrane curvature is recognized by the septin cytoskeleton. J. Cell Biol. *213*, 23–32.

Cannon, K.S., Woods, B.L., and Gladfelter, A.S. (2017). The unsolved problem of how cells sense micron-scale curvature. Trends Biochem. Sci. *42*, 961–976.

Cherepanov, P.P., and Wackernagel, W. (1995). Gene disruption in *Escherichia coli*: TcR and KmR cassettes with the option of Flp-catalyzed excision of the antibiotic-resistance determinant. Gene *158*, 9–14.

Dalebroux, Z.D., Edrozo, M.B., Pfuetzner, R.A., Ressl, S., Kulasekara, B.R., Blanc, M.P., and Miller, S.I. (2015). Delivery of cardiolipins to the *Salmonella* outer membrane is necessary for survival within host tissues and virulence. Cell Host Microbe *17*, 441–451.

Datsenko, K.A., and Wanner, B.L. (2000). One-step Inactivation of chromosomal genes in *Escherichia coli* K-12 using PCR products. Proc. Natl. Acad. Sci. U S A *97*, 6640–6645.

Dolat, L., and Spiliotis, E.T. (2016). Septins promote macropinosome maturation and traffic to the lysosome by facilitating membrane fusion. J. Cell Biol. *214*, 517–527.

Dowler, S., Kular, G., and Alessi, D.R. (2002). Protein lipid overlay assay. Sci. STKE *2002*, https://doi.org/10.1126/stke.2002.129.pl6.

Formal, S.B., Kent, T.H., May, H.C., Palmer, A., Falkow, S., and LaBrec, E.H. (1966). Protection of monkeys against experimental shigellosis with a living attenuated oral polyvalent dysentery vaccine. J. Bacteriol. *92*, 17–22.

Gray, R.D.M., Beerli, C., Pereira, P.M., Scherer, K.M., Samolej, J., Bleck, C.K.E., Mercer, J., and Henriques, R. (2016). Virusmapper: open-source nanoscale mapping of viral architecture through super-resolution microscopy. Sci. Rep. *6*, 29132.

Guzman, L.M., Belin, D., Carson, M.J., and Beckwith, J. (1995). Tight regulation, modulation, and high-level expression by vectors containing the arabinose P(BAD) promoter. J. Bacteriol. *177*, 4121–4130.

Haglund, C.M., and Welch, M.D. (2011). Host-pathogen interactions: pathogens and polymers: microbe-host interactions illuminate the cytoskeleton. J. Cell Biol. *195*, 7–17.

Hartwell, L.H. (1971). Genetic control of the cell division cycle in yeast. iv. genes controlling bud emergence and cytokinesis. Exp. Cell Res. *69*, 265–276.

Kawai, F., Shoda, M., Harashima, R., Sadaie, Y., Hara, H., and Matsumoto, K. (2004). Cardiolipin domains in *Bacillus subtilis* marburg membranes. J. Bacteriol. *186*, 1475–1483.

Kieser, K.J., and Kagan, J.C. (2017). Multi-receptor detection of individual bacterial products by the innate immune system. Nat. Rev. Immunol. *17*, 376–390.

Krokowski, S., and Mostowy, S. (2016). Investigation of septins using infection by bacterial pathogens. Methods Cell Biol. *136*, 117–134.

Landgraf, D., Okumus, B., Chien, P., Baker, T.A., and Paulsson, J. (2012). Segregation of molecules at cell division reveals native protein localization. Nat. Methods *9*, 480–482.

Li, P., Jiang, W., Yu, Q., Liu, W., Zhou, P., Li, J., Xu, J., Xu, B., Wang, F., and Shao, F. (2017). Ubiquitination and degradation of GBPs by a *Shigella* effector to suppress host defence. Nature *551*, 378–383.

Mavrakis, M., Azou-Gros, Y., Tsai, F.-C., Alvarado, J., Bertin, A., Iv, F., Kress, A., Brasselet, S., Koenderink, G.H., and Lecuit, T. (2014). Septins promote F-actin ring formation by crosslinking actin filaments into curved bundles. Nat. Cell Biol. *16*, 322–334.

Mavrakis, M., Tsai, F.C., and Koenderink, G.H. (2016). Purification of recombinant human and *Drosophila* septin hexamers for TIRF assays of actin-septin filament assembly. Methods Cell Biol. *136*, 199–220.

McCarthy, R.R., Mazon-Moya, M.J., Moscoso, J.A., Hao, Y., Lam, J.S., Bordi, C., Mostowy, S., and Filloux, A. (2017). Cyclic-di-GMP regulates lipopolysaccharide modification and contributes to *Pseudomonas aeruginosa* immune evasion. Nat. Microbiol. *2*, 17027.

Mostowy, S., and Cossart, P. (2012). Septins: the fourth component of the cytoskeleton. Nat. Rev. Mol. Cell Biol. *13*, 183–194.

Mostowy, S., and Shenoy, A. (2015). The cytoskeleton in cell-autonomous immunity: structural determinants of host defence. Nat. Rev. Immunol. *15*, 559–573.

Mostowy, S., Bonazzi, M., Hamon, M.A., Tham, T.N., Mallet, A., Lelek, M., Gouin, E., Demangel, C., Brosch, R., Zimmer, C., et al. (2010). Entrapment of intracytosolic bacteria by septin cage-like structures. Cell Host Microbe *8*, 433–444.

Ogawa, M., Yoshimori, T., Suzuki, T., Sagara, H., Mizushima, N., and Sasakawa, C. (2005). Escape of intracellular *Shigella* from autophagy. Science *307*, 727–731.

Oliver, P.M., Crooks, J.A., Leidl, M., Yoon, E.J., Saghatelian, A., and Weibel, D.B. (2014). Localization of anionic phospholipids in *Escherichia coli* cells. J. Bacteriol. *196*, 3386–3398.

Pagliuso, A., Tham, T.N., Stevens, J.K., Lagache, T., Persson, R., Salles, A., Olivo-Marin, J.-C., Oddos, S., Spang, A., Cossart, P., et al. (2016). A role for septin 2 in Drp1-mediated mitochondrial fission. EMBO Rep. *17*, 1–16.

Parsot, C. (2009). *Shigella* type III secretion effectors: how, where, when, for what purposes? Curr. Opin. Microbiol. *12*, 110–116.

Pfanzelter, J., Mostowy, S., and Way, M. (2018). Septins suppress the release of vaccinia virus from infected cells. J. Cell Biol. *217*, 2911–2929.

Pogliano, J., Pogliano, K., Weiss, D.S., Losick, R., and Beckwith, J. (1997). Inactivation of FtsI inhibits constriction of the FtsZ cytokinetic ring and delays the assembly of FtsZ rings at potential division sites. Proc. Natl. Acad. Sci. U S A *94*, 559–564.

Randow, F., MacMicking, J.D., and James, L.C. (2013). Cellular self-defense: how cell-autonomous immunity protects against pathogens. Science *340*, 701–706.

Rossi, R.M., Yum, L., Agaisse, H., and Payne, S.M. (2017). Cardiolipin synthesis and outer membrane localization are required for *Shigella flexneri* virulence. Mbio *8*, https://doi.org/10.1128/mBio.01199-17.

Saarikangas, J., and Barral, Y. (2011). The emerging functions of septins in metazoans. EMBO Rep. *12*, 1118–1126.

Schlosser-Silverman, E., Elgrably-Weiss, M., Rosenshine, I., Kohen, R., and Altuvia, S. (2000). Characterization of Escherichia coli DNA lesions generated within J774 macrophages. J. Bacteriol. *182*, 5225–5230.

Sirianni, A., Krokowski, S., Lobato-Márquez, D., Buranyi, S., Pfanzelter, J., Galea, D., Willis, A., Culley, S., Henriques, R., Larrouy-Maumus, G., et al. (2016). Mitochondria mediate septin cage assembly to promote autophagy of *Shigella*. EMBO Rep. *17*, 1–15.

Spiliotis, E.T. (2018). Spatial effects—site-specific regulation of actin and microtubule organization by septin GTPases. J. Cell Sci. *131*, 1–10.

Torraca, V., and Mostowy, S. (2016). Septins and bacterial infection. Front. Cell Dev. Biol. *4*, 127.

Valdivia, R.H., and Falkow, S. (1996). Bacterial genetics by flow cytometry: rapid isolation of salmonella typhimurium acid-inducible promoters by differential fluorescence induction. Mol. Microbiol. *22*, 367–378.

Wandel, M.P., Pathe, C., Werner, E.I., Ellison, C.J., Boyle, K.B., von der Malsburg, A., Rohde, J., and Randow, F. (2017). GBPs inhibit motility of *Shigella flexneri* but are targeted for degradation by the bacterial ubiquitin ligase IpaH9.8. Cell Host Microbe *22*, 507–518.

Welch, M.D., and Way, M. (2013). Arp2/3-mediated actin-based motility: a tail of pathogen abuse. Cell Host Microbe *14*, 242–255.

STAR★METHODS

KEY RESOURCES TABLE

REAGENT or RESOURCE	SOURCE	IDENTIFIER
Antibodies		
Rabbit polyclonal anti-SEPT7	IBL	Cat#18991; RRID: AB_1630825
Rabbit polyclonal anti-LC3	Abcam	Cat#ab48394; RRID: AB_881433
Rabbit polyclonal anti-p62	MBL	Cat#PM045; RRID: AB_1279301
Mouse monoclonal anti-His	Thermo Fisher Scientific	Cat#MA1-21315; RRID: AB_557403
Mouse monoclonal anti-p62	BD Biosciences	Cat#610832; RRID: AB_398151
Mouse monoclonal anti-GAPDH	Abcam	Cat#ab8245; RRID: AB_2107448
Bacterial and Virus Strains		
Escherichia coli DH5α	Thermo Fisher Scientific	Cat#18265017
Pseudomonas aeruginosa PAK::tn7gfp	(McCarthy et al., 2017)	N/A
Shigella flexneri M90T Serotype 5a	(Mostowy et al., 2010)	N/A
S. flexneri M90T Serotype 5a GFP	(Mostowy et al., 2010)	N/A
S. flexneri M90T Serotype 5a *ΔclsΔymdCΔybhO*	This study	N/A
S. sonnei 53G	(Formal et al., 1966)	N/A
Staphylococcus aureus RN6390-GFP	Laboratory of Tracy Palmer	N/A
Chemicals, Peptides, and Recombinant Proteins		
L-α-phosphatidylethanolamine-N-(lissamine rhodamine B sulfonyl)	Sigma-Aldrich	Cat# 810146C
Nonfunctionalised silica microspheres 1.05 μm	Bang Laboratories	Cat#SS04000
Nonfunctionalised silica microspheres 4.89 μm	Bang Laboratories	Cat#SS05003
Experimental Models: Cell Lines		
HeLa Human cervix carcinoma epithelial cell line	ATCC	CCL-2
HeLa pLVX-puro SEPT6-GFP N-terminal fusion	(Sirianni et al., 2016)	N/A
HeLa pLVX-puro SEPT6-RFP N-terminal fusion	(Pfanzelter et al., 2018)	N/A
U-2 OS Human osteosarcoma epithelial cell line	ATCC	HTB-96
Oligonucleotides		
Sequences of oligonucleotides are listed in Table S1.	This manuscript	N/A
Recombinant DNA		
pBAD18	(Guzman et al., 1995)	N/A
pBAD18 FtsZ-GFP	This study	N/A
pBAD18 SulA	This study	N/A
pDHL584	(Landgraf et al., 2012)	N/A
pFPV25.2	(Valdivia and Falkow, 1996)	N/A
pKD3	(Datsenko and Wanner, 2000)	N/A
pKD4	(Datsenko and Wanner, 2000)	N/A
pKD46	(Datsenko and Wanner, 2000)	N/A
pCP20	(Datsenko and Wanner, 2000)	N/A

(*Continued on next page*)

Continued

REAGENT or RESOURCE	SOURCE	IDENTIFIER
pSA10	(Schlosser-Silverman et al., 2000)	N/A
pSA10 FtsZ-GFP	This study	N/A
pSA11	(Schlosser-Silverman et al., 2000)	N/A
Software and Algorithms		
Prism	Graphpad software	https://www.graphpad.com/
FIJI	NIH	https://imagej.net/ImageJ
ICY	Institut Pasteur	http://icy.bioimageanalysis.org/
Huygens Professional software	SVI	N/A
ZEN	Zeiss	N/A
Benchling	N/A	https://benchling.com

CONTACT FOR REAGENT AND RESOURCE SHARING

Further information and requests for resources and reagents should be directed to and will be fulfilled by the Lead Contact, Serge Mostowy (serge.mostowy@lshtm.ac.uk).

EXPERIMENTAL MODEL AND SUBJECT DETAILS

Bacterial Strains

Bacterial strains used in this study are listed in the Key Resources Table. *E. coli* DH5-α were grown in Lysogeny Broth (LB) broth and plates. *Shigella* were grown on (Trypto-Casein-Soy) TCS plates containing 0.1% (*w/v*) Congo red dye or in TCS broth. *Pseudomonas aeruginosa* GFP (kindly provided by A. Filloux; McCarthy et al., 2017) and *Staphylococcus aureus* GFP (kindly provided by T. Palmer) were grown on TCS plates or in TCS broth. Selection markers were used at the indicated concentrations: carbenicillin (100 μg/ml); kanamycin (50 μg/ml); chloramphenicol (30 μg/ml).

Cell Lines

HeLa (ATCC CCL, female), HeLa SEPT6-GFP, HeLa SEPT6-RFP (kindly provided by M. Way; Pfanzelter et al., 2018) or U-2 OS (ATCC HTB-96, female) cells were cultured in DMEM (GIBCO) supplemented with 10 % fetal bovine serum (FBS) at 37°C and 5% CO_2. For selection, 1 μg/ml puromycin was added to the culturing media of HeLa SEPT6-GFP and HeLa SEPT6-RFP cells.

METHOD DETAILS

Bacterial Growth

Shigella were grown overnight and diluted to a starting OD_{600} of 0.01. Samples were prepared in triplicates in a 96-well plate. OD_{600} was measured every 30 min for 15 h at 37°C with shaking using a microplate reader (TECAN Infinite M200 Pro).

Construction of Deletion Mutants

Primers used in this study were designed using Benchling (https://benchling.com) and are listed in Table S1. *S. flexneri* mutants Δ*cls*, Δ*ymdC* and Δ*ybhO* were created using λ-Red-mediated recombination (Datsenko and Wanner, 2000). For Δ*cls* and Δ*ymdC*, a PCR product was generated by using pKD4 and primers SK-105 (fw) and SK-106 (rev) or SK-113 (fw) and SK-114 (rev), respectively. For Δ*ybhO*, a PCR product was generated using pKD3 and primers SK-107 (fw) and SK-108 (rev). PCR products were electroporated into *S. flexneri* producing λ-Red recombinase (Datsenko and Wanner, 2000) and recombinants were selected by kanamycin (Δ*cls*::*kan* and Δ*ymdC*::*kan*) or chloramphenicol (Δ*ybhO*::*cat*) resistance. Double and triple mutants were created by removing the kanamycin cassette in Δ*cls* using pCP20 (Cherepanov and Wackernagel, 1995), followed by subsequent bacteriophage P1 transduction of the Δ*ymdC*::*kan* and the Δ*ybhO*::*cat* alleles. All strains were verified via PCR. The abbreviation ΔCL is used throughout the manuscript for Δ*cls*Δ*ymdC*Δ*ybhO*.

Plasmid Construction

Constructed plasmids were confirmed by sequencing. Plasmid encoding *sulA* from pBAD18 was constructed by amplifying *sulA* from *S. flexneri* chromosomal DNA using primers SK-101 (fw) and SK-102 (rev). The resulting PCR product was digested with *SalI* and *EcoRI* restriction enzymes and introduced in the *SalI/EcoRI* site of plasmid pBAD18.

Plasmid encoding *ftsZ-gfp* from pSA10 was constructed by amplifying *ftsZ* from *S. flexneri* chromosomal DNA using primers SK-45 (fw) and SK-20 (rev) and by amplifying a monomeric superfolder *gfp* from plasmid pDHL584 (Landgraf et al., 2012) using primers

SK-19 (fw) and SK-54 (rev). The DNA fragment containing *gfp* gene was fused to the C-terminus of the *ftsZ* encoding DNA fragment using Gibson assembly. The *ftsZ-gfp* DNA fragment was digested with *SalI* and *EcoRI* restriction enzymes and introduced into the *SalI*/*EcoRI* restriction site of plasmid pBAD18 (Guzman et al., 1995).

Cardiolipin Detection Using MALDI-TOF MS

Bacteria were grown to stationary phase and heat killed at 90°C for 1 h. After three washes in water, bacteria were diluted to a final concentration of 10^4-10^5 bacteria per μl. To ionize cardiolipin, 0.5 μl matrix [10 mg / ml 2,5-dihydroxybenzoic acid in 90:10 chloroform / methanol (*v/v*)] and 0.5 μl bacteria solution were applied on the target and dried gently under a stream of air. MALDI-TOF MS analysis was performed using the reflectron mode on a 4800 Proteomics Analyzer (with TOF-TOF Optics Applied Biosystems). Samples were analysed in the negative ion mode operated at 20 kV with an extraction delay time of 20 ns. The negative mass spectrum was scanned between *m/z* 1000 and 3000 and MS data were analysed using Data Explorer 4.9 (Applied Biosystems).

Protein-Lipid Overlay Assays

SEPT2/6/7 was purified as previously described in Mavrakis et al. (2014). Purification of SEPT2, SEPT6 or SEPT9 was performed according to Bai et al. (2013). Membrane lipid-strips were purchased from Echelon Biosciences (Salt Lake City, Utah) and *E. coli* cardiolipin was purchased from Avanti Polar Lipids (Alabaster, Alabama). Membrane lipid strips were used according to the manufacturer's instructions with 50 mM Tris, pH 7.5, 150 mM NaCl, 0.1% Tween20, 3% Fatty-acid-free BSA used as blocking buffer. Protein-lipid overlay assays were carried out according to Dowler et al. (2002) using 200 nM septins. The binding of His-SEPT2 and His-SEPT9 was visualised using primary antibodies against His and secondary 680-conjugates goat anti mouse antibodies. The fluorescent signal was detected using the Odyssey imager (Licor Biosciences).

Liposome Flotation Assay

Stationary phase *S. flexneri* WT or ΔCL were centrifuged, washed and resuspended in chloroform/methanol (1:1, v/v) and lipids were extracted three times. To generate small unilamellar vesicles (SUVs), lipids were dried completely under a light nitrogen stream and rehydrated in liposome buffer (50 mM Tris pH 8.0, 50 mM KCl) for 60 min at 65°C and 700 rpm. Lipids were frozen and thawed five times in liquid nitrogen and passed through a mini-extruder (Avanti Polar Lipids) at least 20 times using a 0.1 μm pore filter on a heat block at 65°C. To test for septin binding, 8 mg/ml SUVs were mixed with 0.5 μM purified SEPT2/6/7 trimer in 150 μl liposome buffer containing 1 mM $MgCl_2$. Samples were incubated at 25°C and 500 rpm for 1 h, transferred into a polycarbonated ultracentrifuge tube (Beckman Coulter) and mixed with 100 μl liposome buffer containing 75% sucrose. Samples were gently overlaid with liposome buffer containing 25% sucrose and 50 μl liposome buffer and centrifuged at 48,000 rpm in a TLA-120.2 rotor for 1 h at 20°C. Equal volumes of top and bottom fractions were taken, mixed with Laemmli buffer and run on a 12% SDS-PAGE gel. Samples were blotted and incubated with anti-His antibody followed by peroxidase-conjugated goat anti-mouse (Dako). Membranes were scanned using the ChemiDoc Touch Imaging System (Biorad).

Septin Recruitment to Supported Lipid Bilayer Microspheres

Septin recruitment to supported lipid bilayer microspheres was performed as described in Bridges et al. (2016). In brief, SUVs were made from total bacterial lipid extracts adding <0.1% L-α-phosphatidylethanolamine-*N*-(lissamine rhodamine B sulfonyl) (Avanti Polar Lipids). SUVs were adsorbed onto nonfunctionalised silica microspheres (1.05 or 4.89 μm mean diameter, rounded in text for simplicity; Bang Laboratories) and 8 mg/ml lipids were mixed with 1 or 5 μm beads in 80 μl at 500 rpm and 25°C for 1h. Beads were centrifuged for 30 s and washed 4x in liposome buffer containing 50 mM $MgCl_2$. SEPT2/6/7 was fluorescently labelled using Alexa Fluor 488 NHS Ester as previously described (Mavrakis et al., 2016). After labelling, septins were dialysed against storage buffer (50 mM Tris, pH 8.0, 300 mM KCl, 5 mM $MgCl_2$ and 5 mM DTT). Coated beads were mixed with labelled SEPT2/6/7 to gain a final septin concentration of 50 nM. Beads were imaged on 2 % agarose pads using an Axiovert Z1 driven by ZEN software (Carl Zeiss MicroImaging). 'Septin recruitment' was calculated according to Bridges et al. (2016) using ICY.

Antibodies

Rabbit polyclonal antibodies used were anti-SEPT7 (IBL 18991), anti-LC3 (ab48394) and anti-p62 (MBL PM045). Mouse monoclonal antibodies used were His (Thermo Scientific MA1-21315), p62 (BD 610832) and GAPDH (ab8245). Secondary antibodies used were Alexa Fluor 680-conjugated goat anti-mouse, Alexa 488-, 555- or 647-conjugated donkey anti-rabbit and Alexa Fluor 647-conjugated donkey anti-mouse (Molecular Probes). F-actin was labelled with Alexa 488- or 647-phalloidin (Molecular Probes).

For the immunoblots, total cellular extracts were prepared using Laemmli buffer and blotted with the primary antibodies anti-GAPDH and anti-p62 followed by peroxidase-conjugated goat anti-mouse (Dako) and anti-rabbit secondary antibodies (Dako). GAPDH was used as a loading control. Proteins were run on 10 % SDS polyacrylamide gels.

Bacterial Infection of Epithelial Cells

Cells for fixed microscopy were plated (1 - 5 x 10^5) on glass coverslips in 6-well plates (Thermo Scientific) and used for experiments 24 - 48 h later. HeLa cells for time-lapse microscopy were grown (5 x 10^5) on MatTek glass-bottom dishes (MatTek corporation) and infected 24 h later.

For *Shigella* infections, bacteria were grown to OD_{600} ~0.6 and added to HeLa cells at an MOI of 100. Samples were centrifuged at 110 g for 10 min at 21°C and incubated for 30 min at 37°C and 5% CO_2. Cells were washed with PBS and incubated in 50 μg/ml gentamicin-containing media. Where indicated, arabinose or antibiotics were added at the same time as gentamicin (40 min after infection to allow bacteria to invade host cells and escape to the cytosol). FtsZ-GFP or SulA production was induced with 0.2 % or 1 % arabinose, respectively. Erythromycin was used at 14 μM, trimethoprim was used at 70 μM and cephalexin was used at 2 mg / ml. 3 μg / ml FM4-64X or 0.1 mM Isopropyl β-D-1-thiogalactopyranoside (IPTG) was added 30 min prior to fixation. 10 μM chloroquine was added 15 h before infection. Drugs were kept in the media throughout the infection process. For gentamicin protection assays, intracellular bacteria were extracted after 1 h and 4 h 40 min from infected HeLa cells using 0.1% Triton X-100 (Krokowski and Mostowy, 2016). Lysates were serially diluted and plated on LB agar.

For *Pseudomonas* infections, bacteria were grown to OD_{600} ~2.5, diluted in DMEM to an MOI of 10 and added to HeLa cells. Samples were centrifuged at 1000 rpm for 5 min and incubated for 30 min at 37°C and 5% CO_2. Cells were washed with PBS and incubated in 400 μg/ml gentamicin-containing media for 3 h 25 min for fixed and 1 h for time-lapse microscopy.

For *Staphylococcus* infections, bacteria were grown to OD_{600} ~0.3, washed with PBS and diluted in DMEM to an MOI of 20. Bacteria were added to HeLa cells, centrifuged for 5 min at 1000 rpm and incubated for 30 min at 37°C and 5% CO_2. Cells were washed with PBS and incubated in DMEM containing 100 μg/ml gentamicin and 10 μg/ml lysostaphin for 20 min at 37°C. Infected cells were washed with PBS and incubated for further 35 min in DMEM containing 100 μg/ml gentamicin for time-lapse microscopy.

Fluorescence Microscopy of Infected Cells

Infected HeLa cells were fixed in 4 % paraformaldehyde (PFA) for 15 min at room temperature (RT), washed with PBS and quenched for 10 min in 0.05 M ammonium chloride at RT. Afterwards cells were permeabilised for 5 min with 0.1% Triton-X-100 at RT and stained for fluorescence microscopy. For the LC3 antibody, cells were fixed and permeabilised in 100% ice-cold methanol. Incubation with primary antibodies was performed in PBS for 1 h 30 min at RT and incubation with secondary antibodies and Phalloidin was performed in PBS for 45 min at RT. Cells were incubated for 10 min in 1 μg / ml DAPI and mounted in aqua polymount mounting medium (Polyscience).

Fixed cells were imaged on a fluorescence-inverted microscope Axiovert 200M (Carl Zeiss) driven by Volocity software (Parkin Elmer) (Figures 3A and S3B), confocal microscope LSM 710 (Carl Zeiss) driven by ZEN 2010 software (Figure 1A), Elyra PS.1 microscope driven by ZEN software (Carl Zeiss) (Figures S1F–S1H and 3D) or Axiovert Z1 driven by ZEN Blue 2.3 software (Carl Zeiss) (all remaining Figures). For time-lapse microscopy, samples were imaged in FluoroBrite medium (Life Technologies) supplied with 5% FCS, 4 mM L-glutamine and 50 μg/ml gentamicin at the Axiovert Z1 at 37°C and 5% CO_2 or at the confocal microscope LSM 710 at 37°C for 3 h.

For microscopy of *Shigella* FtsZ-GFP in broth, bacteria were grown to exponential phase and FtsZ-GFP production was induced for further 2 h using 0.1 % L-arabinose. Where indicated, *Shigella* strains (WT, FtsZ-GFP) were treated with 3 μg / ml FM4-64X for 30 min and / or 1 μg / ml DAPI for 10 min before analysis.

Microscopy images were quantified using Z-stack image series taking 5 to 15 slides every 0.2 – 0.4 μm. Image processing was performed using Fiji or Icy (http://icy.bioimageanalysis.org) and deconvolution was performed using Huygens deconvolution software or ZEN software. Bacterial cell length and width were measured manually using the plugin 'Coli-inspector' for FIJI. ICY was used to generate fluorescence profiles (FIP) using the plugin 'Intensity Profile' and to generate 3D images using the plugins 'Stereo 3D Canvas (VTK)' and '3D Rotation'.

Structured Illumination Microscopy

For fixed SIM, U-2 OS cells (1 x 10^5) were seeded on high precision glass coverslips (Carl Roth) in 6-well-plates (Thermo Scientific) and used 48 h later for infection with S. *flexneri* FtsZ-GFP. After 3 h 40 min of infection, cells were fixed, quenched and immunolabelled with primary or secondary antibodies in PBS containing 0.1% Triton-X-100 and 5% horse serum. After a second fixation in 4% PFA for 15 min, cells were imaged in 100 mM beta-mercaptoethylamine pH 7.5. SIM imaging was performed using Plan-Apochromat 63x/1.4 oil DIC M27 objective, in an Elyra PS.1 microscope (Zeiss). Images were acquired using 5 phase shifts and 3 grid rotations and processed using the ZEN software (2012, version 8.1.6.484, Carl Zeiss). For channel alignment, a multi-coloured bead slide was imaged using the same image acquisition settings. For single particle analysis (Gray et al., 2016), the septal region and the long axis of the bacterial cell were manually selected in the FtsZ channel. All bacterial cells were automatically aligned using these two references and the resulting stacks were averaged to create population representative models.

QUANTIFICATION AND STATISTICAL ANALYSIS

Statistical analysis was performed in Excel (Microsoft) and GraphPad Prism (v6.0, La Jolla, USA). Host cells dying from bacterial load were excluded from analysis. Unless otherwise indicated, data represent the mean ± standard error of the mean (SEM) from at least 3 independent experiments. Student's t-test (unpaired, two-tailed), one-way ANOVA or Kruskal-Wallis were used to test for statistical significance, with $p < 0.05$ considered as significant. Fold changes were calculated from each independent experiment and the average ± SEM are given in the text. All statistical details including statistical tests, significance, exact value of n bacterial cells and definition of centre and dispersion can be found in the figure legends.

Cell Host & Microbe, Volume *24*

Supplemental Information

Septins Recognize and Entrap Dividing

Bacterial Cells for Delivery to Lysosomes

Sina Krokowski, Damián Lobato-Márquez, Arnaud Chastanet, Pedro Matos Pereira, Dimitrios Angelis, Dieter Galea, Gerald Larrouy-Maumus, Ricardo Henriques, Elias T. Spiliotis, Rut Carballido-López, and Serge Mostowy

Cell Host & Microbe

Supplemental Information

Septins Recognise Dividing Bacterial Cells for Delivery to the Lysosome

Sina Krokowski, Damián Lobato-Márquez, Arnaud Chastanet, Pedro Matos Pereira, Dimitrios Angelis, Dieter Galea, Gerald Larrouy-Maumus, Ricardo Henriques, Elias T. Spiliotis, Rut Carballido-López, Serge Mostowy

Figure S1. Septins Recognise Micron-Scale Bacterial Curvature, Related to Figure 1.

(A) Illustrating the engineering of a plasmid-encoded (pBAD18), arabinose-controlled FtsZ-monomeric superfolder green fluorescent protein (GFP) fusion in *S. flexneri*.

(B) Localisation of FtsZ-GFP in *S. flexneri* in broth with respect to membrane (FM4-64X) and DNA (DAPI). Scale bar = 1 µm.

(C) Length and width of WT and FtsZ-GFP overproducing *Shigella*. Graph shows median and whiskers (min to max) from n = 600 bacterial cells from 3 independent experiments. Student's t-test, ns $p > 0.05$.

(D) Growth of *Shigella* WT, Vector (empty pBAD18) and FtsZ-GFP was determined by measuring the optical density (OD_{600}) every 30 min for 15 h using a microplate reader. Graph shows mean OD_{600} ± SEM from 7 independent experiments. Student's t-test on last time point, ns $p > 0.05$.

(E) HeLa cells were infected with *Shigella* WT, Vector (empty pBAD18) or FtsZ-GFP and the intracellular bacterial load (CFU / mL) was determined 1 h and 4 h 40 min post infection. Invasion of host cells and intracellular survival is not affected by pBAD18 or FtsZ-GFP. Graph shows mean CFU / mL ± SEM from 3 independent experiments. Student's t-test, ns $p > 0.05$.

(F) *S. flexneri* FtsZ-GFP infected U-2 OS cells at 3 h 40 min post infection. Single-particle analysis of 28 SIM images of *Shigella*-septin cages with SEPT7 localising to FtsZ. Scale bar = 1 µm. Inset images highlight a conserved septin ring at the bacterial division site. Fluorescent intensity profile (FIP) was taken of the dotted line along the midline of the cell in the inset image and normalised from 0 to 1.

(G) Representative three-dimensional SIM images (top) and diagram (bottom) of a SEPT7 ring surrounding the bacterial Z-ring.

(H) Time-lapse SIM of *S. sonnei*-mCherry infected SEPT6-GFP HeLa cells at 1 h 40 min post infection imaged every 2 min. White arrowhead indicates SEPT6-GFP recruitment to the bacterial division site. Scale bar = 1 µm.

Figure S2. Cardiolipin Promotes Septin Recruitment to *Shigella* Membrane Curvature, Related to Figure 2.

(A) Diagram illustrating CL synthesis and localisation in *Shigella*. The three enzymes catalysing the formation of CL are highlighted in purple. CDP-DAG, CDP-diacylglycerol; PGP, phosphatidylglycerophosphate; PS, phosphatidylserine; PG, phosphatidylglycerol; PE, phosphatidylethanolamine; CL, cardiolipin. CL is synthesized in the inner membrane and gets transported to the outer membrane via PbgA (Dong et al., 2016).

(B) Membrane lipid strips were incubated with 200 nM SEPT9 like in Figure 3A.

(C). Negative ion mode MALDI-TOF MS spectra showing distinct cardiolipin peaks in *S. flexneri* WT (purple), which are lost in ΔCL.

(D) Growth of *S. flexneri* WT or ΔCL was determined by measuring the optical density (OD_{600}) every 30 min for 15 h using a microplate reader. Graph shows mean OD_{600} ± SEM from 3 independent experiments. Student's t-test, ns $p > 0.05$.

(E) HeLa cells were infected with *S. flexneri* WT or ΔCL for 3 h 40 min. Graph represents mean % ± SEM of *S. flexneri* polymerising actin (including actin tails and clouds). Values from n = 4945 bacterial cells from 3 independent experiments. Student's t-test, ns $p > 0.05$.

(F) HeLa cells were infected with *S. flexneri* ΔCL for 3 h 40 min, showing cardiolipin-deficient *Shigella* polymerise actin and are not entrapped in SEPT7 cages. Scale bar = 1 µm.

Figure S3. Septins Entrap Actively Dividing Bacteria, Related to Figure 3.

(A-C) Untreated (CTRL), erythromycin (EM) treated or trimethoprim (TMP) treated *S. flexneri* x-light GFP infected HeLa cells at 3 h 40 min post infection. Graph in (A) shows mean % ± SEM of metabolically active *S. flexneri* (i.e. expressing GFP) (Sirianni et al., 2016). Graph in (C) shows mean % ± SEM of actin-polymerising bacteria in CTRL,

EM- or TMP-treated cells. Values from n = 5215 bacterial cells from 3 independent experiments. Student's t-test, ns $p > 0.05$. Representative images showing *S. flexneri* recruiting actin (white arrowheads) (B). Scale bars = 5 µm.

(D) HeLa cells were infected with *S. flexneri* SulA for 3 h 40 min, FM4-64X was added 30 min prior to fixation and samples were stained for endogenous SEPT7. Inset image highlights SEPT7 cage-like structure around filamentous *S. flexneri*. Scale bar = 1 µm.

(E) SEPT6-GFP HeLa cells were infected with *S. flexneri* for 40 min, cephalexin was added for further 45 min and samples were imaged every 2 min. Video frames show septin recruitment to invaginated, filamentous bacteria before cephalexin can fully act.

Figure S4. Septin Cages Inhibit Bacterial Cell Division via Autophagy and Lysosome Fusion, Related to Figure 4.

(A) Whole-cell lysates of untreated (CTRL) or chloroquine treated (CQ) HeLa cells were immunoblotted for p62. GAPDH was used as a loading control.

(B) Untreated (CTRL) or chloroquine treated (CQ) HeLa cells were immunolabelled for p62 and stained for DAPI. Scale bars = 5 µm.

Figure S1. Septins Recognise Micron-Scale Bacterial Curvature.

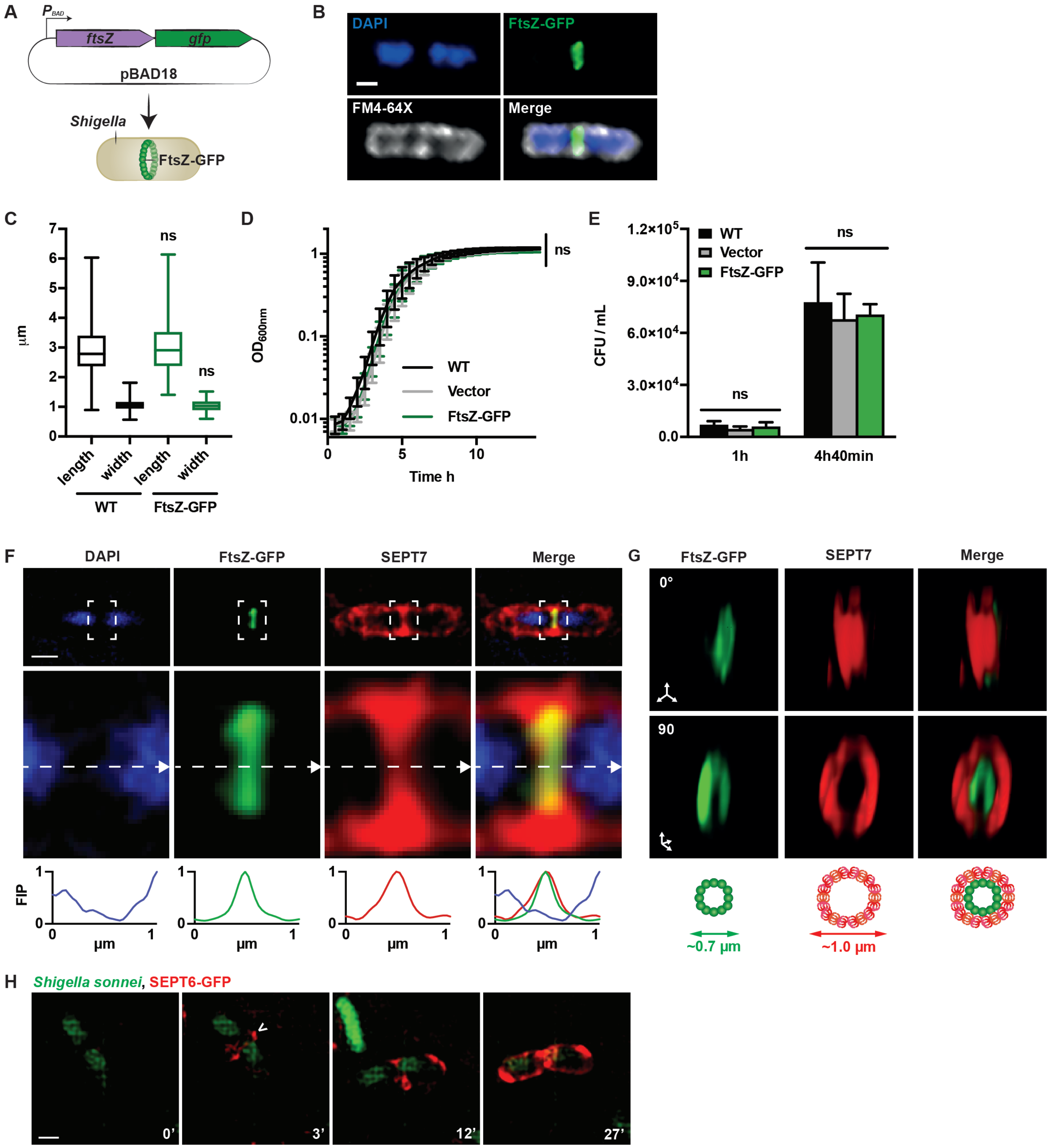

Figure S2. Cardiolipin Promotes Septin Recruitment to *Shigella* Membrane Curvature.

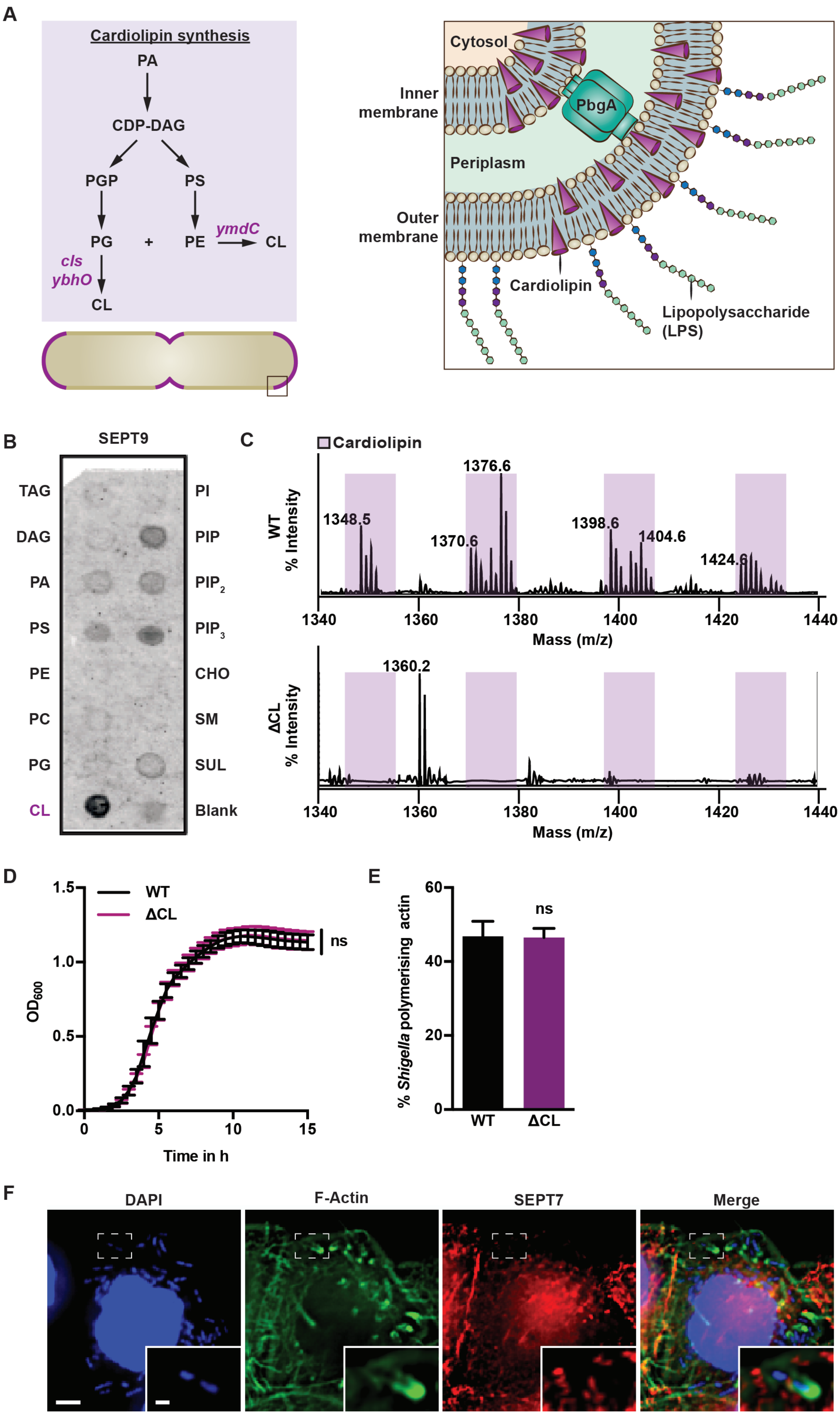

Figure S3. Septin Cages Entrap Actively Dividing Bacteria.

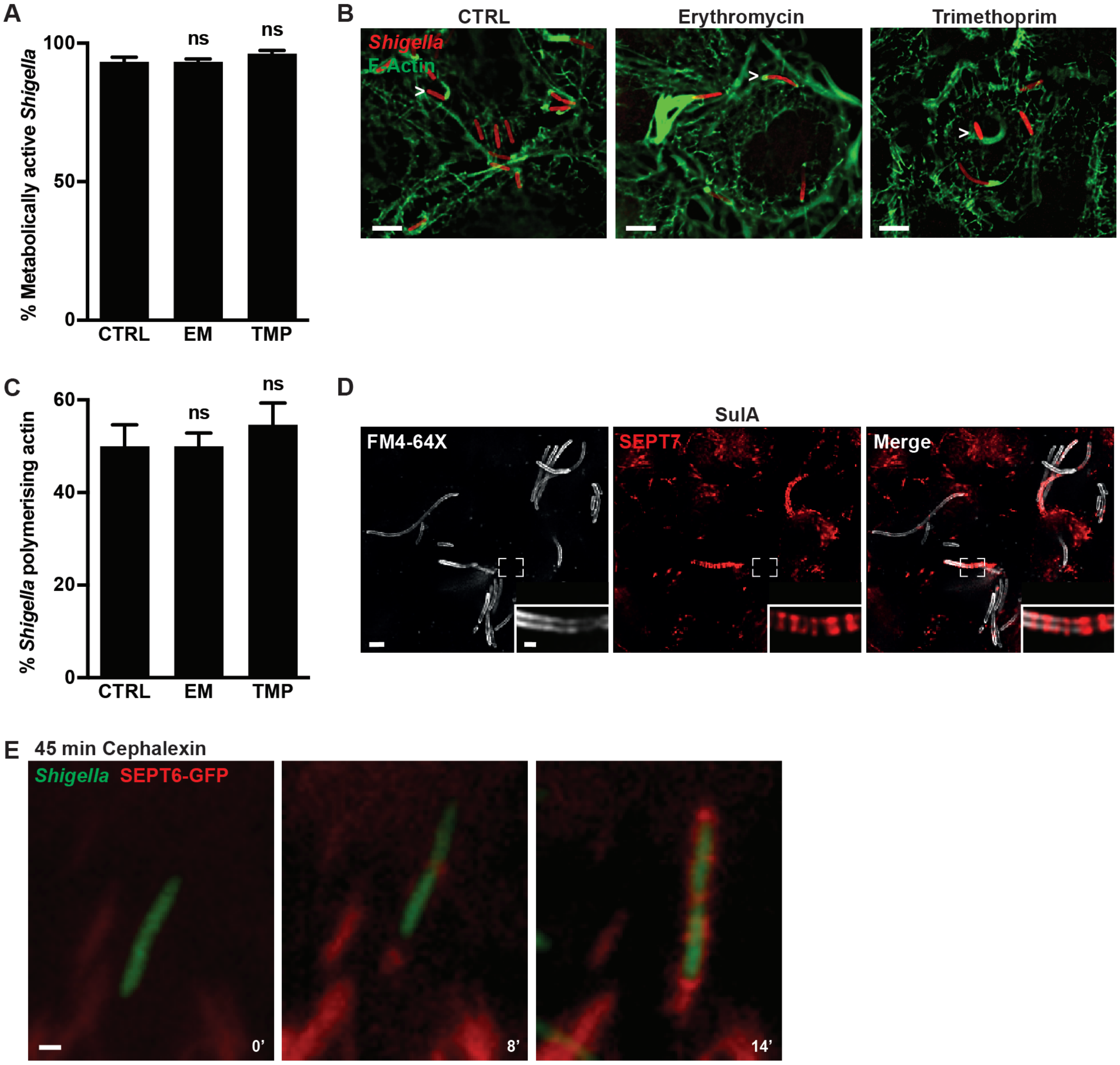

Figure S4. Septin Cages Inhibit Bacterial Cell Division via Autophagy and Lysosome Fusion.

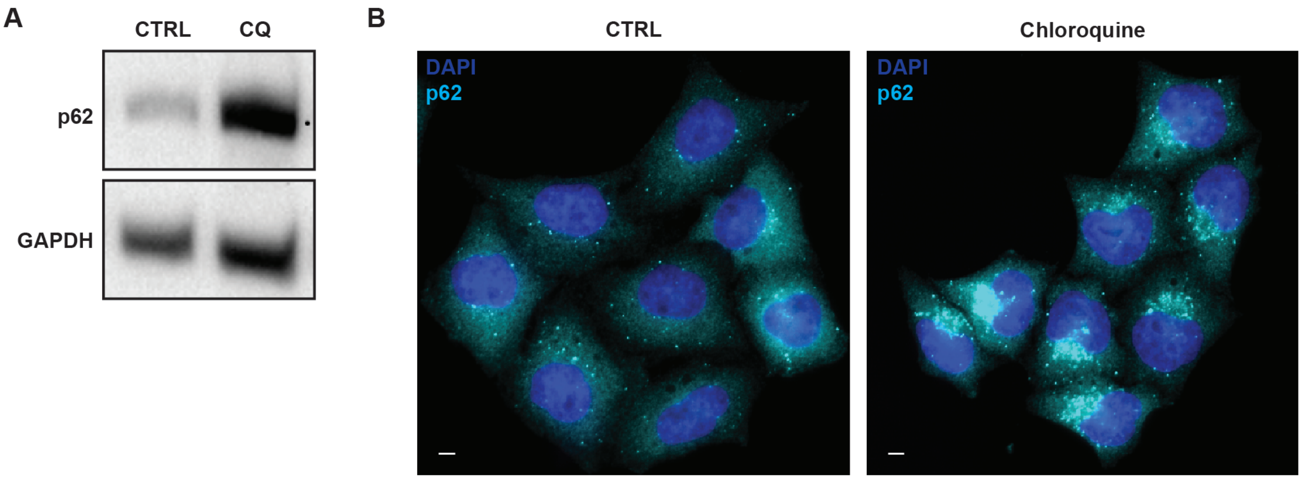

Table S1. Primers used in this study. Related to STAR Methods sections 'Construction of Deletion Mutants' and 'Plasmid construction'.

Primer	DNA sequence
SK-19	CCAGCATTCCTACGTAAGCAAGCTGATAGTAAAGGTGAAGAACTGTTCACCG
SK-20	CACCGGTGAACAGTTCTTCACCTTTACTATCAGCTTGCTTACGTAGGAATGC
SK-45	CTGGTCTCGAATTCATATGTTTGAACCAATGGAACTTACC
SK-54	GTATGTCGACGCTTATTTGTAGAGTTCATCCATGCCG
SK-101	GTATGTCGACGCACTTAATGATACAAATTAGAGTGAA
SK-102	CGATGAATTCATATGTACACTTCAGGCTATGC
SK-105	AGTCAGGCGATTGTTTAGATCCATATCCATAGTCACTACCTGTTTAACCTGTGTAG GCTGGAGCTGCTTC
SK-106	CCCCACTTCCGTTCTACTCCGCTTCATGTTAACTACTCTATGCAATAACACATATG AATATCCTCCTTAGT
SK-107	CGAGATTCAGTGACAAACTGAGCGGATCGAGATTACTGGACCCTACTGTCATGGG AATTAGCCATGGTCC
SK-108	ATGAAATGTAGCTGGCGCGAAGGCAATAAGATCCAGTTGCTGGAAAACGGTGTGT AGGCTGGAGCTGCTTC
SK-113	CACTGTGTTCGCAACATCCCGGTCAGTGTGGTCTTTTCCCTCTGGAGAAAGTGTA GGCTGGAGCTGCTTC
SK-114	TTACAATAACCATTCCACGGGCAATATCGACGCCAGTCTGACCATAACCCCATATG AATATCCTCCTTAGT
